# Dynamic Risk Measures for Processes via Backward Stochastic Differential Equations Associated with Lévy Processes

**DOI:** 10.3390/e23060741

**Published:** 2021-06-11

**Authors:** Liangliang Miao, Zhang Liu, Yijun Hu

**Affiliations:** 1 School of Mathematics and Statistics, Wuhan University, Wuhan 430072, China; yjhu.math@whu.edu.cn; 2 School of Computer and Information Engineering, Jiangxi Agricultural University, Nanchang 330045, China; liuzhang@whu.edu.cn

**Keywords:** dynamic risk measures for processes, dynamic convex risk measures, dynamic coherent risk measures, backward stochastic differential equations, lévy processes, teugel’s martingales

## Abstract

In this paper, we study the dynamic risk measures for processes induced by backward stochastic differential equations driven by Teugel’s martingales associated with Lévy processes (BSDELs). The representation theorem for generators of BSDELs is provided. Furthermore, the time consistency of the coherent and convex dynamic risk measures for processes is characterized by means of the generators of BSDELs. Moreover, the coherency and convexity of dynamic risk measures for processes are characterized by the generators of BSDELs. Finally, we provide two numerical examples to illustrate the proposed dynamic risk measures.

## 1. Introduction

Let (Ω,F,P) be a probability space and T>0 be a fixed terminal time. Let $\left\{B_{t},0\leq t<\infty \right\}$ and $\left\{L_{t},0\leq t<\infty \right\}$ be two mutually independent processes defined on (Ω,F,P), where $\left\{B_{t},0\leq t<\infty \right\}$ is a one-dimensional Brownian motion and $\left\{L_{t},0\leq t<\infty \right\}$ is a R-valued Lévy process corresponding to a standard Lévy measure ν satisfying the following conditions:

(i) $\int_{R}\left(1\wedge y^{2}\right)\nu \left(dy\right)<\infty$,
(ii) $\int_{{\left(-\varepsilon ,\varepsilon \right)}^{c}}e^{\lambda |y|}\nu \left(dy\right)<\infty$, for some λ>0 and for every ε>0.

Let $F=\{F_{t},t\geq 0\}$ be the natural filtration generated by $\left\{B_{t},0\leq t<\infty \right\}$ and $\left\{L_{t},0\leq t<\infty \right\}$.

Throughout this paper, we consider the following integral equation:

(1) $$\begin{array}{c} Y_{t}=\xi +\int_{t}^{T}g\left(s,Y_{s},Z_{s},K_{s}\right)ds-\int_{t}^{T}Z_{s}dB_{s}-\sum_{i=1}^{\infty }\int_{t}^{T}K_{s}^{\left(i\right)}dH_{s}^{\left(i\right)},\;\;\;t\in \left[0,T\right], \end{array}$$

where the terminal value ξ is a given $F_{T}$ -measurable square integrable random variable, g(·) is a given map, and $H_{t}^{\left(i\right)}$ is the orthonormalized Teugel’s martingale of order *i* associated with the Lévy process $\left\{L_{t},0\leq t<\infty \right\}$. The above equation is called backward stochastic differential equations associated with Lévy processes (BSDELs) introduced by Bahlali et al. [1]. When Equation (1) is independent of Teugel’s martingales, then Equation (1) is reduced to the following form:

(2) $$\begin{array}{c} Y_{t}=\xi +\int_{t}^{T}g\left(s,Y_{s},Z_{s},K_{s}\right)ds-\int_{t}^{T}Z_{s}dB_{s},\;\;\;t\in \left[0,T\right], \end{array}$$

which is the classical backward stochastic differential equations (BSDEs) introduced by Pardoux and Peng [2] first. Pardoux and Peng [2] proved that there exists a unique adapted and square integrable solution of the BSDE (2) under uniform Lipschitz condition on *g*. BSDELs can be seen as a natural generalization of BSDEs. Nualart and Schoutens [3] provided a martingale representation theorem associated with Lévy processes. Furthermore, Nualart and Schoutens [4] extended the classical BSDEs to BSDELs and established the existence and uniqueness of solutions for the BSDEL (1) which is independent of Brownian motion. For more studies on BSDELs, see El Otmani [5,6], Ren et al. [7], Ren and El Otmani [8], and the references therein.

Briand et al. [9] first studied the representation theorem for generators of BSDEs under the continuous assumption on the generators with respect to *t* and $E[\sup_{t\in [0,T]}|g\left(t,0,0\right){|}^{2}<\infty ]$. Jiang [10] obtained the representation theorem for Lipschitz generators of BSDEs. Zhang and Fan [11] provided a representation theorem for generators of BSDEs with infinite time intervals and linear growth generators. For more studies on the representation theorem for generators of BSDEs, we refer to Song et al. [12], Xiao and Fan [13], Zheng and Li [14], Wu and Zhang [15], and the references therein. In this paper, we are concerned with representation theorem for generators of the BSDELs (1). For this issue, to our best knowledge, there is no reference available in the literature.

Risk measures have been extensively researched in finance and in the insurance industry such as the adjustment of life insurance rates. To quantify the riskiness of financial positions, Artzner et al. [16,17] introduced the concept of coherent risk measure by proposing the theory of axiomatic system of capital requirements. By weakening coherence axioms, Föllmer and Schied [18] and, independently, Frittelli and Rosazza Gianin [19] introduced convex risk measures. Their work attracts many researcher’s interest. For example, see Delbaen [20], Cheridito et al. [21,22], Riedel [23], Rosazza Gianin [24], Detlefsen and Scandolo [25], Klöppel and Schweizer [26], Delbaen et al. [27], Acciaio et al. [28], Föllmer and Schied [29], Song et al. [30], and the references therein.

BSDEs have become a popular tool for studying dynamic risk measures since Peng [31] investigated BSDEs and g-expectations. For instance, El Karoui et al. [32] studied dynamic risk measures for random variables via BSDEs. Jiang [33] established the one-to-one relationship between the generators BSDEs and the corresponding dynamic risk measures for random variables. Penner and Réveillac [34] established a link between risk measures for processes and BSDEs and studied the corresponding time-consistent dynamic risk measures for processes induced by BSDEs. Xu [35] studied multidimensional dynamic convex risk measures induced by conditional g-expectations. Ji et al. [36] provided some time-consistent dynamic risk measures for processes via BSDEs, and established the one-to-one relationship between the generators BSDEs and the corresponding dynamic risk measures for processes. A essential property for dynamic risk measures is time-consistency (see Bion-Nadal [37,38]). These time-consistent dynamic risk measures are constructed by BSDEs where the financial positions are random variables at some terminal time. However, time-inconsistent preference is realistic in financial markets. For example, see Yong [39], Wang and Shi [40], and Agram [41].

In this paper, we study dynamic risk measures induced by BSDELs. In a financial market, jump dynamics, which might be caused by policy interference, natural accidents, and so on, indeed exist. For instance, a stock’s price and its return show abnormal and sharp volatility. Thus, investors can be risk-averse and master the best time of those jump dynamics if they have sufficient awareness. Therefore, the processes of stock price and its return can be modeled by BSDELs (1). Based on the above consideration, we construct dynamic risk measures by means of BSDELs (1). First, the representation theorem for generators of BSDELs is provided. Second, the time-consistency of the coherent and convex dynamic risk measures for processes is characterized by means of the generators of BSDELs. Moreover, the coherency and convexity of dynamic risk measures for processes are characterized by the generators of BSDELs. Finally, we provide two numerical examples to illustrate the proposed dynamic risk measures. The obtained results extends the results of Briand et al. [9], Jiang [10], Penner and Réveillac [34], and Ji et al. [36].

The rest of the paper is organized as follows. In Section 2, we briefly state some preliminaries including the definitions of time-consistent dynamic convex and coherent risk measures for processes and some results on BSDELs. The definition of dynamic risk measures for processes induced by BSDELs is also provided in Section 2. In Section 3, our main results are presented, that is, the coherency and convexity of dynamic risk measures for processes are characterized by the generators of BSDELs, and the representation theorem for generators of BSDELs is provided. Section 4 contains all the proofs of the main results of this paper. We provide two numerical examples to illustrate the proposed dynamic risk measures in Section 5. Finally, conclusions are summarized.

## 2. Preliminaries

### 2.1. Notations of Dynamic Risk Measures for Processes

For any positive integer *n* and $z\in R^{n}$, |z| denotes its Euclidean norm. For any t∈[0,T], we introduce the following spaces.

- L${}^{2}\left(\Omega ,F_{t},P\right)$ is the space of random variables ξ which are $F_{t}$-measurable with $E[|\xi {|}^{2}]<\infty$.
- L${}^{\infty }\left(\Omega ,F_{t},P\right)$ is the space of random variables ξ which are $F_{t}$-measurable and essentially bounded.
- $H_{T}^{2}$ is the space of ($F_{t}$)-progressively measurable processes Z:Ω×[0,T]→R such that
  $${∥Z∥}_{H_{T}^{2}}^{2}=E\left[\int_{0}^{T}{\left|Z_{s}\right|}^{2}ds\right]<\infty .$$
- $S_{T}^{2}$ is the space of ($F_{t}$)-progressively measurable and *càdlàg* processes Y:Ω×[0,T]→R such that
  $${∥Y∥}_{S_{T}^{2}}^{2}=E\left[\underset{t\in [0,T]}{\sup}{\left|Y_{t}\right|}^{2}\right]<\infty .$$
- $R^{\infty }$ is the space of ($F_{t}$)-progressively measurable and *càdlàg* processes φ:Ω×[0,T]→R such that
  $${∥\varphi ∥}_{R^{\infty }}={∥\underset{t\in [0,T]}{\sup}\left|\varphi_{t}\right|\;∥}_{\infty }<\infty .$$
- $\ell^{2}$ is the space of real valued sequences ${\left(x_{n}\right)}_{n\geq 0}$ such that
  $${∥x∥}_{\ell^{2}}^{2}=\sum_{n=1}^{\infty }{\left|x_{n}\right|}^{2}<\infty .$$
- $P^{2}\left(\ell^{2}\right)$ is the space of predictable processes K taking values in $\ell^{2}$ such that
  $${∥K∥}_{P^{2}\left(\ell^{2}\right)}^{2}=E\left[\int_{0}^{T}{∥K_{s}∥}_{\ell^{2}}^{2}ds\right]=\sum_{i=1}^{\infty }E\left[\int_{0}^{T}{\left|K_{s}^{\left(i\right)}\right|}^{2}ds\right]<\infty .$$
- $E^{2}$ is the Banach space of processes $\left(Y,Z,K\right)\in S_{T}^{2}\times H_{T}^{2}\times P^{2}\left(\ell^{2}\right)$ under the following norm
  $${∥(Y,Z,K)∥}_{E^{2}}^{2}=E\left[\underset{t\in [0,T]}{\sup}|Y_{t}{|}^{2}+\int_{0}^{T}|Z_{s}{|}^{2}ds+\int_{0}^{T}{∥K_{s}∥}_{\ell^{2}}^{2}ds\right].$$

For the convenience of the reader, we introduce the concept of related time-consistent dynamic risk measures for processes, see Cheridito et al. [22], Penner and Réveillac [34], and Ji et al. [36].

For 0≤t≤s≤T, we define the projection $\pi_{t,s}:=R^{\infty }\to R^{\infty }$ as

$$\pi_{t,s}{\left(X\right)}_{r}=1_{\left[t,T\right]}\left(r\right)X_{r\wedge s},r\in \left[0,T\right],$$

$$R_{t,s}^{\infty }:=\pi_{t,s}\left(R^{\infty }\right)\;and\;R_{t}^{\infty }:=\pi_{t,T}\left(R^{\infty }\right).$$

On a general level, a conditional risk measure $\rho_{t}$ is any map from $R_{t}^{\infty }$ to $L^{\infty }\left(\Omega ,F_{t},P\right)$. $\rho_{t}$ can be described as a risk assessment at time t, which is taken into account the information available up to this time. For t∈[0,T], the map $\rho_{t}$ has the following usual axioms for all $X,Y\in R_{t}^{\infty }$.

(A) (Conditional cash invariance) For all $m\in L^{\infty }\left(\Omega ,F_{t},P\right)$,
  $$\rho_{t}\left(X+m1_{\left[t,T\right]}\right)=\rho_{t}\left(X\right)-m.$$
(B) (Monotonicity) $\rho_{t}\left(X\right)\geq \rho_{t}\left(Y\right),\;\;\mathrm{if}\;X\leq Y.$
(C) (Subadditivity) $\rho_{t}\left(X+Y\right)\leq \rho_{t}\left(X+Y\right).$
(D) (Conditional positive homogeneity) $\rho_{t}\left(\lambda X\right)=\lambda \rho_{t}\left(X\right),\;\;\;\;\forall \lambda \in L^{\infty }\left(\Omega ,F_{t},P\right),\lambda \geq 0.$
(E) (Conditional convexity) For all $\lambda \in L^{\infty }\left(\Omega ,F_{t},P\right)$, λ∈[0,1],
  $$\rho_{t}\left(\lambda X+(1-\lambda )Y\right)\leq \lambda \rho_{t}\left(X\right)+\left(1-\lambda \right)\rho_{t}\left(Y\right).$$
(F) (Normalization) $\rho_{t}\left(0\right)=0.$

**Definition** **1.** *A map $\rho_{t}:R_{t}^{\infty }\to L^{\infty }\left(\Omega ,F_{t},P\right)$ for t∈[0,T] is called a conditional coherent risk measure for processes, if it satisfies (A), (B), (C), and (D).*

**Definition** **2.** *A map $\rho_{t}:R_{t}^{\infty }\to L^{\infty }\left(\Omega ,F_{t},P\right)$ for t∈[0,T] is called a conditional convex risk measure for processes, if it satisfies (A), (B), (E), and (F).*

A sequence ${\left(\rho_{t}\right)}_{t\in [0,T]}$ is called a dynamic coherent risk measure for processes, if for each t∈[0,T], $\rho_{t}:R_{t}^{\infty }\to L^{\infty }\left(\Omega ,F_{t},P\right)$ is a conditional coherent risk measure for processes.

Similarly, a sequence ${\left(\rho_{t}\right)}_{t\in [0,T]}$ is called a dynamic convex risk measure for processes, if for each t∈[0,T], $\rho_{t}:R_{t}^{\infty }\to L^{\infty }\left(\Omega ,F_{t},P\right)$ is a conditional convex risk measure for processes.

For each $X\in R^{\infty }$, we use the notation

$$\rho_{t}\left(X\right)=\rho_{t}\left(\pi_{t,T}\left(X\right)\right).$$

**Definition** **3.** *A dynamic convex risk measure for processes ${\left(\rho_{t}\right)}_{t\in [0,T]}$ is called time consistent if*

$$\rho_{t}\left(X\right)=\rho_{t}\left(X1_{\left[t,s\right)}-\rho_{s}{\left(X\right)}_{\left[s,T\right]}\right),\;\;\;\;\;X\in R^{\infty },t\in \left[0,T\right],s\in \left[t,T\right].$$

### 2.2. Some Results on BSDELs

Let $L_{t_{-}}=\lim_{s↗t}L_{s}$ and $\Delta L_{t}=L_{t}-L_{t_{-}}$. Following Nualart and Schoutens [3,4], the so-called power jumps of the Lévy process $\left\{L_{t},t\in \left[0,T\right]\right\}$ are given by

$$L_{t}^{\left(1\right)}=L_{t},\;\;\;\;L_{t}^{\left(i\right)}=\sum_{0\leq s\leq t}{\left(\Delta L_{s}\right)}^{i},\;\;\;\;i\geq 2.$$

We denote by ${\left(H^{\left(i\right)}\right)}_{i\geq 1}$ the Teugel’s martingales, associated with the Lévy process $\left\{L_{t},t\in \left[0,T\right]\right\}$, which is a linear combination of the $Y^{\left(j\right)},j=1,\ldots ,i$:

$$H_{t}^{\left(i\right)}=c_{i,i}Y_{t}^{\left(i\right)}+c_{i,i-1}Y_{t}^{\left(i-1\right)}+\ldots +c_{i,1}Y_{t}^{\left(1\right)},$$

where $Y_{t}^{\left(i\right)}=L_{t}^{\left(i\right)}-E\left[L_{t}^{\left(i\right)}\right]=L_{t}^{\left(i\right)}-tE\left[L_{1}^{\left(i\right)}\right]$ for all i≥1. From Nualart and Schoutens [3], we can see that the coefficients $c_{i,k}$ correspond to the orthonormalization of the polynomials $1,x,x^{2},\ldots$ with respect to the measure $u\left(dx\right)=x^{2}\nu \left(dx\right)+\sigma^{2}\delta_{0}\left(dx\right)$. The martingales ${\left(H^{\left(i\right)}\right)}_{i\geq 1}$, also called the orthonormalized ith-power-jump processes, can be chosen to be pairwise strongly orthonormal martingales, and their predictable quadratic variation processes are given by

$${\langle H^{\left(i\right)},H^{\left(j\right)}\rangle }_{t}=\delta_{i,j}t,\;\;\;i\geq 1,j\geq 1.$$

We define $A_{t}^{i,j}$ as

$$A_{t}^{i,j}={\left[H^{\left(i\right)},H^{\left(j\right)}\right]}_{t}-{\langle H^{\left(i\right)},H^{\left(j\right)}\rangle }_{t},\;\;\;\;t\in \left[0,T\right],i\geq 1,j\geq 1,$$

which means that $\left\{A_{t}^{i,j},t\in \left[0,T\right]\right\}$ is a martingale.

We assume that

$${\left[A^{i,j},A^{m,n}\right]}_{t}=0,\;\;\;\;\;\;i\geq 1,j\geq 1,m\geq 1,n\geq 1.$$

For more related results on Teugel’s martingales associated with the Lévy process $\left\{L_{t},t\in \left[0,T\right]\right\}$, see Nualart and Schoutens [3,4].

For simplicity of presentation, we rewrite BSDELs (1) as

(3) $$\begin{array}{c} Y_{t}=\xi +\int_{t}^{T}g\left(s,Y_{s},Z_{s},K_{s}\right)ds-\int_{t}^{T}Z_{s}dB_{s}-\sum_{i=1}^{\infty }\int_{t}^{T}K_{s}^{\left(i\right)}dH_{s}^{\left(i\right)},\;\;\;t\in \left[0,T\right], \end{array}$$

where $\xi \in L^{2}\left(\Omega ,F_{T},P\right)$ and $g\left(t,y,z,k\right):=\Omega \times \left[0,T\right]\times R\times R\times \ell^{2}\to R$ is F-progressively measurable. We introduce some assumptions which will be used in this paper.

(H1) (Integrability) $g\left(\cdot ,0,0,0\right)\in H_{T}^{2}.$
(H2) (Lipschitz condition) There exists a constant $C_{L}>0$ such that
  $$\left|g\left(t,y,z,k\right)-g\left(t,y_{1},z_{1},k_{1}\right)\right|\leq C_{L}\left(|y-y_{1}|+|z-z_{1}|+∥k-k_{1}{∥}_{\ell^{2}}\right),$$
  for any $y,y_{1},z,z_{1}\in R,k,k_{1}\in \ell^{2}.$
(H3) (Normalization) g(t,0,0,0)=0,dP×dt−a.s.
(H4) (Convexity) g is convex in (y,z,k), i.e., for any $\left(y_{1},z_{1},k_{1}\right),\left(y_{2},z_{2},k_{2}\right)\in R^{2}\times \ell^{2},$ λ∈[0,1],
$g\left(t,\lambda y_{1}+\left(1-\lambda \right)y_{2},\lambda z_{1}+\left(1-\lambda \right)z_{2},\lambda k_{1}+\left(1-\lambda \right)k_{2}\right)$
  $$\leq \lambda g\left(t,y_{1},z_{1},k_{1}\right)+\left(1-\lambda \right)g\left(t,y_{2},z_{2},k_{2}\right),\;\;\;\;\;dP\times dt-a.s.$$
(H5) (Subadditivity) g is subadditive in (y,z,k), i.e., for any $\left(y_{1},z_{1},k_{1}\right),\left(y_{2},z_{2},k_{2}\right)\in R^{2}\times \ell^{2}$,
  $$g\left(t,y_{1}+y_{2},z_{1}+z_{2},k_{1}+k_{2}\right)\leq g\left(t,y_{1},z_{1},k_{1}\right)+g\left(t,y_{2},z_{2},k_{2}\right),\;\;\;\;dP\times dt-a.s.$$
(H6) (Positive homogeneity) g is positively homogeneous in (y,z,k), i.e., for any $\left(y,z,k\right)\in R^{2}\times \ell^{2}$, α≥0,
  $$g\left(t,\alpha y,\alpha z,\alpha k\right)=\alpha g\left(t,y,z,k\right),\;\;\;\;dP\times dt-a.s.$$
(H7) (Monotonicity) g is nonincreasing in *y*.

**Remark** **1.** *From Bahlali et al. [1], under the assumptions (H1) and (H2), for any $\xi \in L^{2}\left(\Omega ,F_{T},P\right)$, there exists a unique adapted solution $\left(Y,Z,K\right)\in E^{2}$ of Equation (3).*

**Remark** **2.** *Following from Nualart and Schoutens [3,4], in the case of a Poisson process $\left\{N_{t},0\leq t<\infty \right\}$ with parameter λ>0, we know that all Teugel’s martingales are equal to $N_{t}-\lambda t$, that is, $H_{t}^{\left(1\right)}=\frac{N_{t}-\lambda t}{\sqrt{\lambda }}$ and $H_{t}^{\left(i\right)}=0,i\geq 2$. All orthonormalized ith-power-jump processes, i≥2, are equal to zero in the case of a Brownian motion $\left\{B_{t},0\leq t<\infty \right\}$.*

From Theorem 3.2 of Bahlali et al. [1], we can show the following Proposition 1 without any substantial difficulties. Therefore, we omit its proof here. Meanwhile, the following Proposition 2 is taken from Theorem 3.3 of Bahlali et al. [1].

**Proposition** **1.** *Assume that g satisfies (H1) and (H2). For i=1,2, let the terminal condition $\xi_{i}\in L^{2}\left(\Omega ,F_{T},P\right)$, and let $\left(Y^{i},Z^{i},K^{i}\right)\in E^{2}$ be the solution of Equation (3) corresponding to $\xi =\xi_{1},\xi =\xi_{2}$, respectively. Then, the following estimate holds:*

(4) $$\begin{array}{c} E\left[{\left(\underset{0\leq t\leq T}{\sup}\left|Y_{t}^{1}-Y_{t}^{2}\right|\right)}^{2}\right]\leq CE\left[|\xi_{1}-\xi_{2}{|}^{2}\right], \end{array}$$

*where C is a positive constant.*

**Proposition** **2.** *For i=1,2, assume that $g^{i}$ satisfies (H1) and (H2), and let the terminal condition $\xi_{i}\in L^{2}\left(\Omega ,F_{T},P\right)$. Let $\left(Y^{i},Z^{i},K^{i}\right)\in E^{2}$ be the solution of Equation (3) corresponding to $\xi =\xi_{1},\xi =\xi_{2}$, respectively. We suppose the following conditions hold:*

*(i)*  *$\xi^{1}\geq \xi^{2}$, P-a.s.*
*(ii)*  *$g^{1}\left(s,Y^{2},Z^{2},K^{2}\right)\geq g^{2}\left(s,Y^{2},Z^{2},K^{2}\right)$dP×dt-a.s.*
*(iii)*  *For all i∈N, let ${\tilde{K}}^{\left(i\right)}$ denote the $\ell^{2}$-valued stochastic process such that its i first components are equal to those of $K^{2}$ and its N\{1,2,···,i} last components are equal to those of $K^{1}$. With this notation, we define for i∈N*
  $$\begin{array}{c} \gamma_{s}^{i}\;=\;\left\{\begin{array}{ll} {\left(K_{s}^{1(i)}\;-\;K_{s}^{2(i)}\right)}^{-1}\left(g^{1}\left(t,Y_{s}^{2},Z_{s}^{2},{\tilde{K}}_{s}^{\left(i-1\right)}\right)\;-\;g^{1}\left(t,Y_{s}^{2},Z_{s}^{2},{\tilde{K}}_{s}^{\left(i\right)}\right)\right),\;\; & K_{s}^{1(i)}-K_{s}^{2(i)}\neq 0,\\ 0,\;\; & otherwise, \end{array}\right. \end{array}$$
  *satisfying that $\sum_{i=1}^{\infty }\gamma_{t}^{i}▵H_{t}^{i}>-1.$*
*Then,*
  $$Y_{t}^{1}\geq Y_{t}^{2},\;\;\;\;\;\;t\in \left[0,T\right].$$

**Remark** **3.** *The third condition of comparison Theorem 2 is that we add. Without the additional condition, it does not hold in general for solutions of BSDEs associated with Lévy processes (see the counter-example in Barles et al. [42]). In the proof of Bahlali et al. [1], They actually use this condition.*

In this paper, define the dynamic risk measures for processes ρ by

(5) $$\begin{array}{c} \rho_{t}\left(X\right)=Y_{t}\left(X\right),\;\forall t\in \left[0,T\right],X\in R^{\infty }, \end{array}$$

where *Y* is the first component of the solution $\left(Y_{t}\left(X\right),Z_{t}\left(X\right),K_{t}\left(X\right)\right)$ of the following BSDEL:

(6) $$\begin{array}{c} Y_{t}=-X_{T}+\int_{t}^{T}g\left(s,Y_{s}+X_{s},Z_{s},K_{s}\right)ds-\int_{t}^{T}Z_{s}dB_{s}-\sum_{i=1}^{\infty }\int_{t}^{T}K_{s}^{\left(i\right)}dH_{s}^{\left(i\right)},\;\;\;t\in \left[0,T\right]. \end{array}$$

The following lemma shows the existence and uniqueness of the solution of BSDEL (6) and its proof will be postponed to Section 4.

**Lemma** **1.** *Assume that g satisfies (H1) and (H2). For any $X\in S_{T}^{2}$, there exists a unique adapted solution in $E^{2}$, denoted by $(Y_{t,T}\left(X\right),Z_{t,T}\left(X\right),$$K_{t,T}\left(X\right))$, solving BSDEL (6).*

**Remark** **4.** *For simplicity of the notation, we sometimes denote the solution $(Y_{t,T}\left(X\right),Z_{t,T}\left(X\right),$$K_{t,T}\left(X\right))$ of BSDEL (6) by $(Y_{t}\left(X\right),Z_{t}\left(X\right),$$K_{t}\left(X\right))$. Thanks to the uniqueness of the solution, for each $X\in R^{\infty }$, we have that $Y_{t}\left(X\right)=Y_{t}\left(\pi_{t,T}\left(X\right)\right)$, which is consistent with our notation $\rho_{t}\left(X\right)=\rho_{t}\left(\pi_{t,T}\left(X\right)\right)$. For 0≤t≤s≤T, we also denote by $(Y_{t,s}\left(X\right),Z_{t,s}\left(X\right),$$K_{t,s}\left(X\right))$ the solution of BSDEL (6) on [0,s] at time t. Accordingly, for all $t\in \left[0,s\right],X\in R^{\infty }$, we have that $Y_{t,s}\left(X\right)=Y_{t,s}\left(\pi_{t,s}\left(X\right)\right)$.*

## 3. Main Results

In this section, we will state the main results of this paper. Namely, we will state the connections between the generators of BSDELs and the dynamic risk measures for processes via BSDELs. By a product, we will also give a representation theorem of the generators of BSDELs. Their proofs will be postponed to Section 4.

Before we provide the connections between the generators of BSDELs (3) and the dynamic risk measures for processes via BSDELs, we need to give a representation theorem for generators of BSDELs (3), which will be used in the later. As pointed in the Introduction, the study about representation theorems for generators is an interesting topic and is useful in financial mathematics. The following Theorem 1 is one of the main results of this paper.

**Theorem** **1.** *Assume that g satisfies (H1) and (H2). Let the terminal condition $\xi \in L^{2}\left(\Omega ,F_{T},P\right)$. Denote by $\left(Y_{t}\left(g,T,\xi \right),Z_{t}\left(g,T,\xi \right),K_{t}\left(g,T,\xi \right)\right)$ the solution of Equation (3). Then, for each $\left(t,y,z,k\right)\in \left[0,T\right)\times R^{2}\times \ell^{2},p\in \left[1,2\right)$, the following equality*

(7) $$\begin{array}{c} g\left(t,y,z,k\right)=L^{p}-\lim_{\varepsilon \to 0^{+}}\frac{1}{\varepsilon }\left[Y_{t}\left(g,t+\varepsilon ,y+z\left(B_{t+\varepsilon }-B_{t}\right)+\sum_{i=1}^{\infty }k_{i}\left(H_{t+\varepsilon }^{\left(i\right)}-H_{t}^{\left(i\right)}\right)\right)-y\right] \end{array}$$

*holds true for almost every t∈[0,T). Furthermore, there exists a subsequence ${\left\{n_{m}\right\}}_{m=1}^{\infty }\subset {\left\{n\right\}}_{n=1}^{\infty }$ such that dP×dt-a.s.,*

(8) $$\begin{array}{c} g\left(t,y,z,k\right)=\lim_{m\to \infty }n_{m}\left[Y_{t}\left(g,t+\frac{1}{n_{m}},y+z\left(B_{t+\frac{1}{n_{m}}}-B_{t}\right)+\sum_{i=1}^{\infty }k_{i}\left(H_{t+\frac{1}{n_{m}}}^{\left(i\right)}-H_{t}^{\left(i\right)}\right)\right)-y\right]. \end{array}$$

Now, we are in a position to state the connections between the generators of BSDELs and the dynamic risk measures for processes via BSDELs, which are another main results of this paper.

**Theorem** **2.** *Assume that g satisfies (H1) and (H2). Denote by $(Y_{t}\left(X\right),Z_{t}\left(X\right),$$K_{t}\left(X\right))$ the solution of BSDEL (6) corresponding to $X\in R^{\infty }$. Let ρ be defined as (5). Then,*

*(i)*  *${\left(\rho_{t}\right)}_{t\in [0,T]}$ is a dynamic convex risk measure for processes if and only if g satisfies assumption (H3), (H4), and (H7).*
*(ii)*  *If ${\left(\rho_{t}\right)}_{t\in [0,T]}$ is a dynamic convex risk measure for processes, then ${\left(\rho_{t}\right)}_{t\in [0,T]}$ is time-consistent.*

**Theorem** **3.** *Assume that g satisfies (H1) and (H2). Denote by $(Y_{t}\left(X\right),Z_{t}\left(X\right),$$K_{t}\left(X\right))$ the solution of BSDEL (6) corresponding to $X\in R^{\infty }$. Let ρ be defined as (5). Then,*

*(i)*  *${\left(\rho_{t}\right)}_{t\in [0,T]}$ is a dynamic coherent risk measure for processes if and only if g satisfies assumption (H5), (H6), and (H7).*
*(ii)*  *If ${\left(\rho_{t}\right)}_{t\in [0,T]}$ is a dynamic coherent risk measure for processes, then ${\left(\rho_{t}\right)}_{t\in [0,T]}$ is time-consistent.*

By choosing some specific generators of BSDELs, we construct dynamic risk measures for processes by means of BSDELs.

**Remark** **5.** *Consider $g:\Omega \times \left[0,T\right]\times R\times R\times \ell^{2}\to R$ defined by g(t,y,z,k)=−y+|z|+k. Let $(Y_{t}\left(X\right),Z_{t}\left(X\right),$$K_{t}\left(X\right))$ be the adapted solution of BSDEL (6) corresponding to $X\in S_{T}^{2}$. Let $\rho_{t}\left(X\right)=Y_{t}\left(X\right),\;\forall t\in \left[0,T\right],X\in R^{\infty }.$ Then, ρ is a dynamic coherent risk measure.*

**Remark** **6.** *Consider $g:\Omega \times \left[0,T\right]\times R\times R\times \ell^{2}\to R$ defined by $g\left(t,y,z,k\right)=-y+{2|z|}^{2}+k$ if |z|≤1, and g(t,y,z,k)=−y+3|z|+k−1 if |z|≥1. Let $(Y_{t}\left(X\right),Z_{t}\left(X\right),$$K_{t}\left(X\right))$ be the adapted solution of BSDEL (6) corresponding to $X\in S_{T}^{2}$. Let $\rho_{t}\left(X\right)=Y_{t}\left(X\right),\;\forall t\in \left[0,T\right],X\in R^{\infty }.$ Then, ρ is a dynamic convex risk measure. However, ρ is not a dynamic coherent risk measure.*

## 4. Proofs of Main Results

In this section, we will provide the proof of Lemma 1 and all proofs of the results stated in Section 3.

**Proof** **of** **Lemma** **1.** In order to prove the existence and uniqueness of the solution of BSDEL (6), we define a new function $f^{X}:\Omega \times \left[0,T\right]\times R\times R\times \ell^{2}\to R$ as

(9) $$\begin{array}{c} f^{X}\left(t,y,z,k\right):=g\left(t,y+X\left(s\right),z,k\right), \end{array}$$

where $t\in \left[0,T\right],\;\left(y,z,k\right)\in R\times R\times \ell^{2}.$ It is easy to see that $f^{X}$ satisfies the Lipschitz condition (H2). Therefore, we only need to show that $f^{X}$ satisfies assumption (H1). By using assumption (H2), we obtain for all t∈[0,T],

$$\begin{array}{cc} \int_{0}^{T}{\left|f^{X}\left(s,0,0,0\right)\right|}^{2}ds & =\int_{0}^{T}{\left|g\left(s,X\left(s\right),0,0\right)\right|}^{2}ds\\ & \leq 2C_{L}^{2}\left(\int_{0}^{T}{\left|g\left(s,0,0,0\right)\right|}^{2}ds+\int_{0}^{T}{\left|X\left(s\right)\right|}^{2}ds\right)\\ & \leq 2C_{L}^{2}\left(\int_{0}^{T}{\left|g\left(s,0,0,0\right)\right|}^{2}ds+\underset{s\in [t,T]}{\sup}{\left|X\left(s\right)\right|}^{2}\right). \end{array}$$

Notice that *g* satisfies assumption (H1) and $X\in S_{T}^{2}$. By taking mathematical expectation, we immediately deduce that

$$E\left[\int_{0}^{T}{\left|f^{X}\left(s,0,0,0\right)\right|}^{2}ds\right]<\infty .$$

Thus, $f^{X}$ satisfies assumption (H1).  □

In order to prove Theorem 1, we need to have two additional results. The following Proposition 3 comes from Proposition 2.2 of Jiang [33]. Proposition 4 concerning on a priori estimate for BSDELs is new and needs to be proved.

**Proposition** **3.** *Let q>1 and 1≤p<q. For any ($F_{t}$)-progressively measurable process ψ:Ω×[0,T]→R satisfying $E\left[\int_{0}^{T}{\left|\psi_{s}\right|}^{q}ds\right]<\infty$, the following equality*

$$\psi_{t}=L^{p}-\lim_{\varepsilon \to 0^{+}}\frac{1}{\varepsilon }\int_{t}^{t+\varepsilon }\psi_{s}ds$$

*holds true for almost every t∈[0,T).*

**Proposition** **4.** *Assume that g satisfies (H1) and (H2). Denote by (Y,Z,K) the solution of BSDEL (3) corresponding to $\xi \in L^{2}\left(\Omega ,F_{T},P\right)$. Then we have*

$$\begin{array}{cc} E & \left[\underset{s\in [t,T]}{\sup}e^{\beta s}|Y_{s}{|}^{2}+\int_{t}^{T}e^{\beta s}|Z_{s}{|}^{2}ds+\sum_{i=1}^{\infty }\int_{t}^{T}e^{\beta s}{\left|K_{s}^{\left(i\right)}\right|}^{2}ds|F_{t}\right]\\ & \leq CE\left[e^{\beta T}{\left|\xi \right|}^{2}+{\left(\int_{t}^{T}e^{\frac{\beta s}{2}}\left|g\left(s,0,0,0\right)\right|ds\right)}^{2}|F_{t}\right], \end{array}$$

*where C is a positive constant and $\beta =2C_{L}+4C_{L}^{2}$.*

**Proof.**  By Itô’s formula (see Theorem 32 of Protter [43], Page 78), for any constant β, we have

(10) $$\begin{array}{c} e^{\beta t}|Y_{t}{|}^{2}+\int_{t}^{T}e^{\beta s}|Z_{s}{|}^{2}ds+\sum_{i=1}^{\infty }\int_{t}^{T}e^{\beta s}{\left|K_{s}^{\left(i\right)}\right|}^{2}ds\\ =e^{\beta T}{\left|\xi \right|}^{2}-\int_{t}^{T}\beta e^{\beta s}{\left|Y_{s}\right|}^{2}ds+2\int_{t}^{T}e^{\beta s}Y_{s}g\left(s,Y_{s},Z_{s},K_{s}\right)ds-2\int_{t}^{T}e^{\beta s}Y_{s}Z_{s}dB_{s}\\ \;\;\;\;-2\sum_{i=1}^{\infty }\int_{t}^{T}e^{\beta s}Y_{s}K_{s}^{\left(i\right)}dH_{s}^{\left(i\right)}-\sum_{i=1}^{\infty }\sum_{j=1}^{\infty }\int_{t}^{T}e^{\beta s}K_{s}^{\left(i\right)}K_{s}^{\left(j\right)}dA_{s}^{i,j}. \end{array}$$

Applying the Lipschitz condition (H2) to *g* and then the inequality $ab\leq \frac{a^{2}}{2}+\frac{b^{2}}{2}$, we deduce that

(11) $$\begin{array}{cc} 2 & \int_{t}^{T}e^{\beta s}Y_{s}g\left(s,Y_{s},Z_{s},K_{s}\right)ds\\ & \leq 2\int_{t}^{T}e^{\beta s}\left|Y_{s}\right|\left|g(s,Y_{s},Z_{s},K_{s})\right|ds\\ & \leq 2\int_{t}^{T}e^{\beta s}\left|Y_{s}\right|\left|g(s,Y_{s},Z_{s},K_{s}\right)-g\left(s,0,0,0\right)|ds+2\int_{t}^{T}e^{\beta s}\left|Y_{s}\right||g\left(s,0,0,0\right)|ds\\ & \leq 2C_{L}\int_{t}^{T}e^{\beta s}\left|Y_{s}\right|\left(|Y_{s}\left|+\right|Z_{s}|+∥K_{s}{∥}_{\ell^{2}}\right)ds+2\int_{t}^{T}e^{\beta s}\left|Y_{s}\right||g\left(s,0,0,0\right)|ds\\ & \leq 2C_{L}\int_{t}^{T}e^{\beta s}{\left|Y_{s}\right|}^{2}ds+2C_{L}^{2}\int_{t}^{T}e^{\beta s}{\left|Y_{s}\right|}^{2}ds+\frac{1}{2}\int_{t}^{T}e^{\beta s}{\left|Z_{s}\right|}^{2}ds\\ & \;\;\;+2C_{L}^{2}\int_{t}^{T}e^{\beta s}{\left|Y_{s}\right|}^{2}ds+\frac{1}{2}\int_{t}^{T}e^{\beta s}∥K_{s}{∥}_{\ell^{2}}^{2}ds+2\int_{t}^{T}e^{\beta s}\left|Y_{s}\right||g\left(s,0,0,0\right)|ds\\ & =\left(2C_{L}+4C_{L}^{2}\right)\int_{t}^{T}e^{\beta s}{\left|Y_{s}\right|}^{2}ds+\frac{1}{2}\int_{t}^{T}e^{\beta s}{\left|Z_{s}\right|}^{2}ds+\frac{1}{2}\int_{t}^{T}e^{\beta s}{∥K_{s}∥}_{\ell^{2}}^{2}ds\\ & \;\;\;+2\int_{t}^{T}e^{\beta s}\left|Y_{s}\right||g\left(s,0,0,0\right)|ds. \end{array}$$

Taking $\beta =2C_{L}+4C_{L}^{2}$, from (10) and (11), we have

(12) $$\begin{array}{c} e^{\beta t}{\left|Y_{t}\right|}^{2}+\frac{1}{2}\int_{t}^{T}e^{\beta s}{\left|Z_{s}\right|}^{2}ds+\frac{1}{2}\sum_{i=1}^{\infty }\int_{t}^{T}e^{\beta s}{\left|K_{s}^{\left(i\right)}\right|}^{2}ds\\ \leq e^{\beta T}{\left|\xi \right|}^{2}-2\int_{t}^{T}e^{\beta s}Y_{s}Z_{s}dB_{s}-2\sum_{i=1}^{\infty }\int_{t}^{T}e^{\beta s}Y_{s}K_{s}^{\left(i\right)}dH_{s}^{\left(i\right)}\\ \;\;\;\;-\sum_{i=1}^{\infty }\sum_{j=1}^{\infty }\int_{t}^{T}e^{\beta s}K_{s}^{\left(i\right)}K_{s}^{\left(j\right)}dA_{s}^{i,j}+2\int_{t}^{T}e^{\beta s}\left|Y_{s}\right||g\left(s,0,0,0\right)|ds. \end{array}$$

Therefore, we get

(13) $$\begin{array}{c} e^{\beta t}{\left|Y_{t}\right|}^{2}+\frac{1}{2}E\left[\int_{t}^{T}e^{\beta s}{\left|Z_{s}\right|}^{2}ds|F_{t}\right]+\frac{1}{2}E\left[\sum_{i=1}^{\infty }\int_{t}^{T}e^{\beta s}{\left|K_{s}^{\left(i\right)}\right|}^{2}ds|F_{t}\right]\\ \leq 2E\left[e^{\beta T}{\left|\xi \right|}^{2}+\int_{t}^{T}e^{\beta s}\left|Y_{s}\right||g\left(s,0,0,0\right)|ds|F_{t}\right], \end{array}$$

and

(14) $$\begin{array}{cc} \underset{u\in [t,T]}{\sup}e^{\beta u}{\left|Y_{u}\right|}^{2} & \leq e^{\beta T}{\left|\xi \right|}^{2}+2\int_{t}^{T}e^{\beta s}\left|Y_{s}\right||g\left(s,0,0,0\right)|ds\\ & \;\;\;\;+4\underset{u\in [t,T]}{\sup}\left|\int_{t}^{u}e^{\beta s}Y_{s}Z_{s}dB_{s}\right|+4\underset{u\in [t,T]}{\sup}\left|\sum_{i=1}^{\infty }\int_{t}^{u}e^{\beta s}Y_{s}K_{s}^{\left(i\right)}dH_{s}^{\left(i\right)}\right|\\ & \;\;\;\;+4\underset{u\in [t,T]}{\sup}\left|\sum_{i=1}^{\infty }\sum_{j=1}^{\infty }\int_{t}^{u}e^{\beta s}K_{s}^{\left(i\right)}K_{s}^{\left(j\right)}dA_{s}^{i,j}\right|. \end{array}$$

By Burkholder–Davis–Gundys inequality (see Theorem 48 of Protter [43], Page 193) and then the inequality $ab\leq \frac{a^{2}}{2}+\frac{b^{2}}{2}$, we have

(15) $$\begin{array}{cc} E\left[\underset{u\in [t,T]}{\sup}\left|\int_{t}^{u}e^{\beta s}Y_{s}Z_{s}dB_{s}\right||F_{t}\right] & \leq CE\left[{\left(\int_{t}^{T}e^{2\beta s}{\left|Y_{s}\right|}^{2}{\left|Z_{s}\right|}^{2}ds\right)}^{\frac{1}{2}}|F_{t}\right]\\ & \leq CE\left[{\left(2C\int_{t}^{T}e^{\beta s}{\left|Z_{s}\right|}^{2}ds\right)}^{\frac{1}{2}}{\left(\frac{1}{2C}\underset{s\in [t,T]}{\sup}e^{\beta s}{\left|Y_{s}\right|}^{2}\right)}^{\frac{1}{2}}|F_{t}\right]\\ & \leq C^{2}E\left[\int_{t}^{T}e^{\beta s}{\left|Z_{s}\right|}^{2}ds|F_{t}\right]+\frac{1}{4}E\left[\underset{s\in [t,T]}{\sup}e^{\beta s}{\left|Y_{s}\right|}^{2}|F_{t}\right]. \end{array}$$

Using Burkholder–Davis–Gundys inequality again, we easily deduce that

(16) $$\begin{array}{c} E\left[\underset{u\in [t,T]}{\sup}\left|\sum_{i=1}^{\infty }\sum_{j=1}^{\infty }\int_{t}^{u}e^{\beta s}K_{s}^{\left(i\right)}K_{s}^{\left(j\right)}dA_{s}^{i,j}\right||F_{t}\right]=0. \end{array}$$

Similarly, by Burkholder–Davis–Gundys inequality, ${\left|a+b\right|}^{r}\leq \max\left(1,2^{r-1}\right){\left(|a\right|}^{r}+{\left|b\right|}^{r}),r>0$ and then Jensen’s inequality (see Theorem 19 of Protter [43], Page 11), we have

$$\begin{array}{cc} & E\left[\underset{u\in [t,T]}{\sup}\left|\sum_{i=1}^{\infty }\int_{t}^{u}e^{\beta s}Y_{s}K_{s}^{\left(i\right)}dH_{s}^{\left(i\right)}\right||F_{t}\right]\\ & \;\;\;\;\leq CE\left[{\left(\sum_{i=1}^{\infty }\sum_{j=1}^{\infty }\int_{t}^{T}e^{2\beta s}{\left|Y_{s}\right|}^{2}K_{s}^{\left(i\right)}K_{s}^{\left(j\right)}d{\left[H^{\left(i\right)},H^{\left(j\right)}\right]}_{s}\right)}^{\frac{1}{2}}|F_{t}\right]\\ & \;\;\;\;=CE\left[{\left(\sum_{i=1}^{\infty }\int_{t}^{T}e^{2\beta s}{\left|Y_{s}\right|}^{2}{\left|K_{s}^{\left(i\right)}\right|}^{2}ds+\sum_{i=1}^{\infty }\sum_{j=1}^{\infty }\int_{t}^{T}e^{2\beta s}{\left|Y_{s}\right|}^{2}K_{s}^{\left(i\right)}K_{s}^{\left(j\right)}dA_{s}^{i,j}\right)}^{\frac{1}{2}}|F_{t}\right]\\ & \;\;\;\;\leq CE\left[{\left(\sum_{i=1}^{\infty }\int_{t}^{T}e^{2\beta s}{\left|Y_{s}\right|}^{2}{\left|K_{s}^{\left(i\right)}\right|}^{2}ds\right)}^{\frac{1}{2}}|F_{t}\right]+CE\left[{\left|\sum_{i=1}^{\infty }\sum_{j=1}^{\infty }\int_{t}^{T}e^{2\beta s}{\left|Y_{s}\right|}^{2}K_{s}^{\left(i\right)}K_{s}^{\left(j\right)}dA_{s}^{i,j}\right|}^{\frac{1}{2}}|F_{t}\right] \end{array}$$

(17) $$\begin{array}{cc} \; & \;\;\;\;\leq CE\left[{\left(\sum_{i=1}^{\infty }\int_{t}^{T}e^{2\beta s}{\left|Y_{s}\right|}^{2}{\left|K_{s}^{\left(i\right)}\right|}^{2}ds\right)}^{\frac{1}{2}}|F_{t}\right]+CE{\left[\left|\sum_{i=1}^{\infty }\sum_{j=1}^{\infty }\int_{t}^{T}e^{2\beta s}{\left|Y_{s}\right|}^{2}K_{s}^{\left(i\right)}K_{s}^{\left(j\right)}dA_{s}^{i,j}\right||F_{t}\right]}^{\frac{1}{2}}\\ \; & \;\;\;\;\leq CE\left[{\left(\sum_{i=1}^{\infty }\int_{t}^{T}e^{2\beta s}{\left|Y_{s}\right|}^{2}{\left|K_{s}^{\left(i\right)}\right|}^{2}ds\right)}^{\frac{1}{2}}|F_{t}\right]\\ \; & \;\;\;\;\;\;\;\;+CE{\left[\underset{u\in [t,T]}{\sup}\left|\sum_{i=1}^{\infty }\sum_{j=1}^{\infty }\int_{t}^{u}e^{2\beta s}{\left|Y_{s}\right|}^{2}K_{s}^{\left(i\right)}K_{s}^{\left(j\right)}dA_{s}^{i,j}\right||F_{t}\right]}^{\frac{1}{2}}\\ \; & \;\;\;\;=CE\left[{\left(\sum_{i=1}^{\infty }\int_{t}^{T}e^{2\beta s}{\left|Y_{s}\right|}^{2}{\left|K_{s}^{\left(i\right)}\right|}^{2}ds\right)}^{\frac{1}{2}}|F_{t}\right]\\ \; & \;\;\;\;\leq C^{2}E\left[\sum_{i=1}^{\infty }\int_{t}^{T}e^{\beta s}{\left|K_{s}^{\left(i\right)}\right|}^{2}ds|F_{t}\right]+\frac{1}{4}E\left[\underset{s\in [t,T]}{\sup}e^{\beta s}{\left|Y_{s}\right|}^{2}|F_{t}\right]. \end{array}$$

Therefore, we have

(18) $$\begin{array}{cc} E\left[\underset{s\in [t,T]}{\sup}e^{\beta s}{\left|Y_{s}\right|}^{2}|F_{t}\right] & \leq E\left[e^{\beta T}{\left|\xi \right|}^{2}+2\int_{t}^{T}e^{\beta s}\left|Y_{s}\right||g\left(s,0,0,0\right)|ds|F_{t}\right]\\ & \;\;\;\;+C^{2}E\left[\int_{t}^{T}e^{\beta s}{\left|Z_{s}\right|}^{2}ds|F_{t}\right]+\frac{1}{4}E\left[\underset{s\in [t,T]}{\sup}e^{\beta s}{\left|Y_{s}\right|}^{2}|F_{t}\right]\\ & \;\;\;\;+C^{2}E\left[\sum_{i=1}^{\infty }\int_{t}^{T}e^{\beta s}{\left|K_{s}^{\left(i\right)}\right|}^{2}ds|F_{t}\right]+\frac{1}{4}E\left[\underset{s\in [t,T]}{\sup}e^{\beta s}{\left|Y_{s}\right|}^{2}|F_{t}\right]. \end{array}$$

Finally, combining (13) and (18), there exists a constant C>0 such that

$$\begin{array}{cc} E & \left[\underset{s\in [t,T]}{\sup}e^{\beta s}{\left|Y_{s}\right|}^{2}+\int_{t}^{T}e^{\beta s}{\left|Z_{s}\right|}^{2}ds+\sum_{i=1}^{\infty }\int_{t}^{T}e^{\beta s}{\left|K_{s}^{\left(i\right)}\right|}^{2}ds|F_{t}\right]\\ & \leq CE\left[e^{\beta T}{\left|\xi \right|}^{2}+\int_{t}^{T}e^{\beta s}\left|Y_{s}\right||g\left(s,0,0,0\right)|ds|F_{t}\right]\\ & \leq CE\left[e^{\beta T}{\left|\xi \right|}^{2}+\left(\frac{1}{\sqrt{C}}\underset{s\in [t,T]}{\sup}e^{\frac{\beta s}{2}}\left|Y_{s}\right|\right)\left(\sqrt{C}\int_{t}^{T}e^{\frac{\beta s}{2}}\left|g\left(s,0,0,0\right)\right|ds\right)|F_{t}\right]\\ & \leq CE\left[e^{\beta T}{\left|\xi \right|}^{2}+\frac{C}{2}{\left(\int_{t}^{T}e^{\frac{\beta s}{2}}\left|g\left(s,0,0,0\right)\right|ds\right)}^{2}|F_{t}\right]+\frac{1}{2}E\left[\underset{s\in [t,T]}{\sup}e^{\beta s}{\left|Y_{s}\right|}^{2}|F_{t}\right]. \end{array}$$

Thus, we have completed the proof of this proposition.  □

Based on Propositions 3 and 4, we can prove Theorem 1.

**Proof** **of** **Theorem** **1.** Let us pick ε>0 small enough such that t+ε<T. Suppose that *g* satisfies (H1) and (H2). For any given $\left(t,y,z,k\right)\in \left[0,T\right)\times R^{2}\times \ell^{2}$, we consider the following BSDEL:

(19) $$\begin{array}{cc} Y_{s}^{\varepsilon }= & y+z\left(B_{t+\varepsilon }-B_{t}\right)+\sum_{i=1}^{\infty }k_{i}\left(H_{t+\varepsilon }^{\left(i\right)}-H_{t}^{\left(i\right)}\right)+\int_{s}^{t+\varepsilon }g\left(u,Y_{u}^{\varepsilon },Z_{u}^{\varepsilon },K_{u}^{\varepsilon }\right)du\\ \; & -\int_{s}^{t+\varepsilon }Z_{u}^{\varepsilon }dB_{u}-\sum_{i=1}^{\infty }\int_{s}^{t+\varepsilon }K_{u}^{(i),\varepsilon }dH_{u}^{\left(i\right)},\;\;\;\;\;\;\;s\in \left[0,t+\varepsilon \right]. \end{array}$$

Then, there exists a unique adapted solution in $E^{2}$, denoted by $(Y^{\varepsilon },Z^{\varepsilon },$ $K^{\varepsilon })$, solving BSDEL (19)

For any given $\left(t,y,z,k\right)\in \left[0,T\right)\times R^{2}\times \ell^{2}$ and t<s<t+ε, let us set

$${\tilde{Y}}_{s}^{\varepsilon }:=Y_{s}^{\varepsilon }-\left(y+z\left(B_{s}-B_{t}\right)+\sum_{i=1}^{\infty }k_{i}\left(H_{s}^{\left(i\right)}-H_{t}^{\left(i\right)}\right)\right),\;\;{\tilde{Z}}_{s}:=Z_{s}^{\varepsilon }-z,\;\;{\tilde{K}}_{s}:=K_{s}^{\varepsilon }-k.$$

Applying Itô’s formula to ${\tilde{Y}}_{s}^{\varepsilon }$, we have

(20) $$\begin{array}{cc} {\tilde{Y}}_{s}^{\varepsilon }= & \int_{s}^{t+\varepsilon }g\left(u,{\tilde{Y}}_{u}^{\varepsilon }+y+z\left(B_{u}-B_{t}\right)+\sum_{i=1}^{\infty }k_{i}\left(H_{u}^{\left(i\right)}-H_{t}^{\left(i\right)}\right),{\tilde{Z}}_{u}^{\varepsilon }+z,{\tilde{K}}_{u}^{\varepsilon }+k\right)du\\ \; & -\int_{s}^{t+\varepsilon }{\tilde{Z}}_{u}^{\varepsilon }dB_{u}-\sum_{i=1}^{\infty }\int_{s}^{t+\varepsilon }{\tilde{K}}_{u}^{(i),\varepsilon }dH_{u}^{\left(i\right)},\;\;\;\;\;\;\;s\in \left[t,t+\varepsilon \right]. \end{array}$$

Then, there exists a unique adapted solution in $E^{2}$, denoted by $({\tilde{Y}}^{\varepsilon },{\tilde{Z}}^{\varepsilon },$ ${\tilde{K}}^{\varepsilon })$, solving BSDEL (20).

By Proposition 4, Lipschitz condition (H2) and then Hölder’s inequality (see Proposition 1.3.2 of Zhang [44], Page 13), we deduce that

(21) $$\begin{array}{cc} E & \left[\underset{s\in [t,t+\varepsilon ]}{\sup}{\left|{\tilde{Y}}_{s}^{\varepsilon }\right|}^{2}+\int_{t}^{t+\varepsilon }{\left|{\tilde{Z}}_{s}^{\varepsilon }\right|}^{2}ds+\sum_{i=1}^{\infty }\int_{t}^{t+\varepsilon }{\left|{\tilde{K}}_{s}^{(i),\varepsilon }\right|}^{2}ds|F_{t}\right]\\ \; & \leq Ce^{(2C_{L}+4C_{L}^{2})T}E\left[{\left(\int_{t}^{t+\varepsilon }\left|g\left(u,y+z\left(B_{u}-B_{t}\right)+\sum_{i=1}^{\infty }k_{i}\left(H_{u}^{\left(i\right)}-H_{t}^{\left(i\right)}\right),z,k\right)\right|du\right)}^{2}|F_{t}\right]\\ \; & \leq C_{1}E\left[{\left(\int_{t}^{t\;+\;\varepsilon }\left(\left|g(u,0,0,0)\right|\;+\;|y|\;+\;|z|\;+{\;∥k∥}_{\ell^{2}}+|z\left(B_{u}-B_{t}\right)|\;+\;|k\left(H_{u}-H_{t}\right)|\right)du\right)}^{2}|F_{t}\right]\\ \; & \leq C_{1}E\left[\varepsilon \int_{t}^{t\;+\;\varepsilon }{\left(\left|g(u,0,0,0)\right|\;+\;|y|\;+\;|z|\;+{\;∥k∥}_{\ell^{2}}+|z\left(B_{u}-B_{t}\right)|\;+\;|k\left(H_{u}-H_{t}\right)|\right)}^{2}du|F_{t}\right]\\ \; & \leq 6C_{1}\varepsilon E\left[\int_{t}^{t\;+\;\varepsilon }\left({\left|g(u,0,0,0)\right|}^{2}\;+{\;|y|}^{2}\;+{\;|z|}^{2}\;+{\;∥k∥}_{\ell^{2}}^{2}+|z\left(B_{u}-B_{t}\right){|}^{2}\;+\;{\left|k\left(H_{u}-H_{t}\right)\right|}^{2}\right)du|F_{t}\right], \end{array}$$

where $C_{1}=CC_{L}^{2}e^{(2C_{L}+4C_{L}^{2})T}$ is a positive constant. Therefore, taking the expectation in the previous inequality, we have

(22) $$\begin{array}{cc} E & \left[\underset{s\in [t,t+\varepsilon ]}{\sup}{\left|{\tilde{Y}}_{s}^{\varepsilon }\right|}^{2}+\int_{t}^{t+\varepsilon }{\left|{\tilde{Z}}_{s}^{\varepsilon }\right|}^{2}ds+\sum_{i=1}^{\infty }\int_{t}^{t+\varepsilon }{\left|{\tilde{K}}_{s}^{(i),\varepsilon }\right|}^{2}ds\right]\\ \; & \leq 6C_{1}\varepsilon E\left[\int_{t}^{t\;+\;\varepsilon }\left({\left|g(u,0,0,0)\right|}^{2}\;+{\;|y|}^{2}\;+{\;|z|}^{2}\;+\;{∥k∥}_{\ell^{2}}^{2}\right)du\right]+6C_{1}\varepsilon E\left[\int_{t}^{t\;+\;\varepsilon }{\left|z\left(B_{u}-B_{t}\right)\right|}^{2}du\right]\\ \; & \;\;\;\;+6C_{1}\varepsilon E\left[\int_{t}^{t\;+\;\varepsilon }{\left|k\left(H_{u}-H_{t}\right)\right|}^{2}du\right]. \end{array}$$

By Fubini Theorem, we have

(23) $$\begin{array}{cc} E\left[\int_{t}^{t\;+\;\varepsilon }{\left|z\left(B_{u}-B_{t}\right)\right|}^{2}du\right] & =\int_{t}^{t\;+\;\varepsilon }E\left[|z\left(B_{u}-B_{t}\right){|}^{2}\right]du\\ \; & =\int_{t}^{t\;+\;\varepsilon }z^{2}\left(u-t\right)du\\ \; & =\frac{1}{2}\varepsilon^{2}{\left|z\right|}^{2}\to 0,\;\;\;\;\left(\varepsilon \to 0^{+}\right). \end{array}$$

Applying Fubini Theorem again and then Itô’s formula, we have

(24) $$\begin{array}{cc} \; & E\left[\int_{t}^{t\;+\;\varepsilon }{\left|k\left(H_{u}-H_{t}\right)\right|}^{2}du\right]\\ \; & =\int_{t}^{t\;+\;\varepsilon }E\left[|k\left(H_{u}-H_{t}\right){|}^{2}\right]du\\ \; & =\int_{t}^{t\;+\;\varepsilon }\left\{E\left[2\int_{t}^{u}k\left(H_{s}-H_{t}\right)d\left(k(H_{s}-H_{t})\right)+\int_{t}^{u}d{\left[k\left(H_{\cdot }-H_{t}\right),k\left(H_{\cdot }-H_{t}\right)\right]}_{s}\right]\right\}du\\ \; & =\int_{t}^{t\;+\;\varepsilon }\left\{E\left[\int_{t}^{u}d{\langle k\left(H_{\cdot }-H_{t}\right),k\left(H_{\cdot }-H_{t}\right)\rangle }_{s}\right]\right\}du\\ \; & =\int_{t}^{t\;+\;\varepsilon }\left\{E\left[\int_{t}^{u}{∥k∥}_{\ell^{2}}^{2}ds\right]\right\}du\\ \; & =\frac{1}{2}\varepsilon^{2}{∥k∥}_{\ell^{2}}^{2}\to 0,\;\;\;\;\left(\varepsilon \to 0^{+}\right). \end{array}$$

Combining (23) and (24), and absolute continuity of integral, we obtain

(25) $$\begin{array}{c} \lim_{\varepsilon \to 0^{+}}\frac{1}{\varepsilon }E\left[\underset{s\in [t,t+\varepsilon ]}{\sup}{\left|{\tilde{Y}}_{s}^{\varepsilon }\right|}^{2}+\int_{t}^{t+\varepsilon }{\left|{\tilde{Z}}_{s}^{\varepsilon }\right|}^{2}ds+\sum_{i=1}^{\infty }\int_{t}^{t+\varepsilon }{\left|{\tilde{K}}_{s}^{(i),\varepsilon }\right|}^{2}ds\right]=0 \end{array}$$

Set

$$\begin{array}{cc} M_{t}^{\varepsilon } & :=\frac{1}{\varepsilon }E\left[\int_{s}^{t+\varepsilon }g\left(u,{\tilde{Y}}_{u}^{\varepsilon }+y+z\left(B_{u}-B_{t}\right)+\sum_{i=1}^{\infty }k_{i}\left(H_{u}^{\left(i\right)}-H_{t}^{\left(i\right)}\right),{\tilde{Z}}_{u}^{\varepsilon }+z,{\tilde{K}}_{u}^{\varepsilon }+k\right)du|F_{t}\right]\\ N_{t}^{\varepsilon } & :=\frac{1}{\varepsilon }E\left[\int_{t}^{t+\varepsilon }g\left(u,y,z,k\right)du|F_{t}\right]. \end{array}$$

Taking conditional expectation in the BSDEL (20), we have

(26) $$\begin{array}{cc} \frac{1}{\varepsilon }\left(Y_{t}^{\varepsilon }-y\right) & =\frac{1}{\varepsilon }{\tilde{Y}}_{t}^{\varepsilon }\\ \; & =\frac{1}{\varepsilon }E\left[\int_{t}^{t+\varepsilon }g\left(u,{\tilde{Y}}_{u}^{\varepsilon }+y+z\left(B_{u}-B_{t}\right)+\sum_{i=1}^{\infty }k_{i}\left(H_{u}^{\left(i\right)}-H_{t}^{\left(i\right)}\right),{\tilde{Z}}_{u}^{\varepsilon }+z,{\tilde{K}}_{u}^{\varepsilon }+k\right)du|F_{t}\right]\\ \; & =N_{t}^{\varepsilon }+\left(M_{t}^{\varepsilon }-N_{t}^{\varepsilon }\right). \end{array}$$

By Jensen’s inequality, Hölder’s inequality, Lipschitz condition (H2), and (23)–(25), we have

$$\begin{array}{c} \lim_{\varepsilon \to 0^{+}}E\left[{\left(M_{t}^{\varepsilon }-N_{t}^{\varepsilon }\right)}^{2}\right]\\ =\lim_{\varepsilon \to 0^{+}}\frac{1}{\varepsilon^{2}}E\left[|E\left[\int_{t}^{t+\varepsilon }\left(g(u,{\tilde{Y}}_{u}^{\varepsilon }+y+z\left(B_{u}-B_{t}\right)+\sum_{i=1}^{\infty }k_{i}\left(H_{u}^{\left(i\right)}-H_{t}^{\left(i\right)}\right),{\tilde{Z}}_{u}^{\varepsilon }+z,{\tilde{K}}_{u}^{\varepsilon }+k)\right.\right.\right.\\ \;\;\;\;\left.\left.-g\left(u,y,z,k\right))du|F_{t}\right]{|}^{2}\right]\\ \leq \lim_{\varepsilon \to 0^{+}}\frac{1}{\varepsilon^{2}}E\left[E\left[|\int_{t}^{t+\varepsilon }\left(g(u,{\tilde{Y}}_{u}^{\varepsilon }+y+z\left(B_{u}-B_{t}\right)+\sum_{i=1}^{\infty }k_{i}\left(H_{u}^{\left(i\right)}-H_{t}^{\left(i\right)}\right),{\tilde{Z}}_{u}^{\varepsilon }+z,{\tilde{K}}_{u}^{\varepsilon }+k)\right.\right.\right.\\ \;\;\;\;\left.\left.-g\left(u,y,z,k\right))du{|}^{2}|F_{t}\right]\right] \end{array}$$

(27) $$\begin{array}{cc} \; & \leq \lim_{\varepsilon \to 0^{+}}\frac{5C_{L}^{2}}{\varepsilon }E\left[\int_{t}^{t+\varepsilon }\left(|{\tilde{Y}}_{u}^{\varepsilon }{|}^{2}+|{\tilde{Z}}_{u}^{\varepsilon }{|}^{2}+∥{\tilde{K}}_{u}^{\varepsilon }{∥}_{\ell^{2}}^{2}+|z\left(B_{u}-B_{t}\right){|}^{2}+{\left|k\left(H_{u}-H_{t}\right)\right|}^{2}\right)du\right]\\ \; & =0. \end{array}$$

Using Proposition 3, (H1) and (H2), for any 1≤p<2 and $\left(y,z,k\right)\in R\times R\times \ell^{2}$, we have

(28) $$\begin{array}{c} \lim_{\varepsilon \to 0^{+}}E\left[{\left|N_{t}^{\varepsilon }-g\left(u,y,z,k\right)\right|}^{p}\right]\\ \;\;=\lim_{\varepsilon \to 0^{+}}E\left[{\left|\frac{1}{\varepsilon }E\left[\int_{t}^{t+\varepsilon }g\left(u,y,z,k\right)du|F_{t}\right]-g\left(t,y,z,k\right)\right|}^{p}\right]\\ \;\;=0,\;\;\;a.e.,t\in [0,T). \end{array}$$

Thus, we have completed the proof of the first part of Theorem 1.

By using the relationship between the almost sure convergence and the moment convergence with Fubini’s theorem, we can see that the first part of Theorem 1 directly implies the second part. The proof is complete.  □

The proofs of Theorems 2 and 3 will be decomposed into several steps as outline below.

**Proposition** **5.** *Assume that g satisfies (H1) and (H2). Denote by $(Y_{t}\left(X\right),Z_{t}\left(X\right),$$K_{t}\left(X\right))$ the solution of BSDEL (6) corresponding to $X\in S_{T}^{2}$. Then, the following statements are equivalent:*

*(i)*  *For all t∈[0,T], $Y_{t}\left(0\right)=0$.*
*(ii)*  *g satisfies (H3), that is, g(t,0,0,0)=0,dP×dt−a.s.*

**Proof.**  By the uniqueness of the solution of BSDEL (6), it is clearly seen that (ii) ⇒ (i) holds. Let us prove that (i) ⇒ (ii) holds. Suppose that (i) holds, that is, for all t∈[0,T], $Y_{t}\left(g,T,0\right)=0$. Then, for all s∈[0,t], $Y_{s}\left(g,t,0\right)=0$. Following from Theorem 1, we can see that (ii) holds.  □

For conditional cash invariance and time-consistency of $Y_{t}\left(\cdot \right)$, we have the following result.

**Proposition** **6.** *Assume that g satisfies (H1) and (H2). Denote by $(Y_{t}\left(X\right),Z_{t}\left(X\right),$$K_{t}\left(X\right))$ the solution of BSDEL (6) corresponding to $X\in S_{T}^{2}$. Then, we have the following statements:*

*(i)*  *For any $m\in L^{2}\left(\Omega ,F_{t},P\right),t\in \left[0,T\right]$, $Y_{t}\left(X+m1_{\left[t,T\right]}\right)=Y_{t}\left(X\right)-m$.*
*(ii)*  *If g also satisfies (H3), then ${\left(Y_{t}\left(\cdot \right)\right)}_{t\in [0,T]}$ is time-consistent, i.e.,*
  $$Y_{t}\left(X1_{\left[t,s\right)}-Y_{s}\left(X\right)1_{\left[s,T\right]}\right)=Y_{t}\left(X\right),\;\;\;\;\;$$
  *for all $X\in S_{T}^{2}$ and all t∈[0,T],s∈[t,T].*

**Proof.**  Let us prove that (i) holds. For each $X\in S_{T}^{2}$, $m\in L^{2}\left(\Omega ,F_{t},P\right)$, we consider the following BSDEL:

$$\begin{array}{cc} {\tilde{Y}}_{t}=-X_{T}-m & +\int_{t}^{T}g\left(s,{\tilde{Y}}_{s}+X_{s}+m,{\tilde{Z}}_{s},{\tilde{K}}_{s}\right)ds\\ & -\int_{t}^{T}{\tilde{Z}}_{s}dB_{s}-\sum_{i=1}^{\infty }\int_{t}^{T}{\tilde{K}}_{s}^{\left(i\right)}dH_{s}^{\left(i\right)},\;\;\;t\in \left[0,T\right]. \end{array}$$

Obviously, we have

$$\begin{array}{cc} {\tilde{Y}}_{t}+m=-X_{T} & +\int_{t}^{T}g\left(s,{\tilde{Y}}_{s}+X_{s}+m,{\tilde{Z}}_{s},{\tilde{K}}_{s}\right)ds\\ & -\int_{t}^{T}{\tilde{Z}}_{s}dB_{s}-\sum_{i=1}^{\infty }\int_{t}^{T}{\tilde{K}}_{s}^{\left(i\right)}dH_{s}^{\left(i\right)},\;\;\;t\in \left[0,T\right]. \end{array}$$

Thanks to uniqueness of the solution of BSDEL (6), we get ${\tilde{Y}}_{t}=Y_{t}\left(X+m1_{\left[t,T\right]}\right)$ and ${\tilde{Y}}_{t}+m=Y_{t}\left(X\right)$. Thus, for each $X\in S_{T}^{2}$, $m\in L^{2}\left(\Omega ,F_{t},P\right)$,

$$Y_{t}\left(X+m1_{\left[t,T\right]}\right)=Y_{t}\left(X\right)-m\;\;\;\;\;\;t\in \left[0,T\right].$$

Now we prove that (ii) holds. Suppose that *g* satisfies (H3). To this end, we first prove that

$$Y_{t,T}\left(X\right)=Y_{t,s}\left(X1_{\left[t,s\right)}-Y_{s,T}\left(X\right)1_{\left[s,s\right]}\right),\;\;\;\;\;s\in \left[t,T\right].$$

Let us denote by $\left(Y,Z,K\right)$ the solution of BSDEL (6) corresponding to $X\in S_{T}^{2}$. Following from the uniqueness of the solution of BSDEL (6), we obtain

$$\begin{array}{cc} Y_{t,T}\left(X\right) & =-X_{T}+\int_{t}^{T}g\left(r,Y_{r}+X_{r},Z_{r},K_{r}\right)dr-\int_{t}^{T}Z_{r}dB_{r}-\sum_{i=1}^{\infty }\int_{t}^{T}K_{r}^{\left(i\right)}dH_{r}^{\left(i\right)}\\ =-X_{T}+\int_{s}^{T}g\left(r,Y_{r}+X_{r},Z_{r},K_{r}\right)dr-\int_{s}^{T}Z_{r}dB_{r}-\sum_{i=1}^{\infty }\int_{s}^{T}K_{r}^{\left(i\right)}dH_{r}^{\left(i\right)}\\ \;\;\;\;\;\;\;\;\;\;\;+\int_{t}^{s}g\left(r,Y_{r}+X_{r},Z_{r},K_{r}\right)dr-\int_{t}^{s}Z_{r}dB_{r}-\sum_{i=1}^{\infty }\int_{t}^{s}K_{r}^{\left(i\right)}dH_{r}^{\left(i\right)}\\ =Y_{s,T}\left(X\right)+\int_{t}^{s}g\left(r,Y_{r}+X_{r},Z_{r},K_{r}\right)dr-\int_{t}^{s}Z_{r}dB_{r}-\sum_{i=1}^{\infty }\int_{t}^{s}K_{r}^{\left(i\right)}dH_{r}^{\left(i\right)}\\ =Y_{t,s}\left(X1_{\left[t,s\right)}-Y_{s,T}\left(X\right)1_{\left[s,s\right]}\right). \end{array}$$

Let $\tilde{X}=X1_{\left[t,s\right)}-Y_{s,T}\left(X\right)1_{\left[s,T\right]},t\in \left[0,T\right],s\in \left[t,T\right].$ Then, we get $\tilde{X}\in S_{T}^{2}$. Now, we denote by $\left(\tilde{Y},\tilde{Z},\tilde{K}\right)$ the solution of BSDEL (6) corresponding to $X=\tilde{X}$. Then we have

(29) $$\begin{array}{cc} {\tilde{Y}}_{t} & =Y_{s,T}\left(X\right)+\int_{t}^{s}g\left(r,{\tilde{Y}}_{r}+X_{r},{\tilde{Z}}_{r},{\tilde{K}}_{r}\right)dr-\int_{t}^{s}{\tilde{Z}}_{r}dB_{r}-\sum_{i=1}^{\infty }\int_{t}^{s}{\tilde{K}}_{r}^{\left(i\right)}dH_{r}^{\left(i\right)}\\ & \;\;\;\;-Y_{s,T}\left(X\right)+Y_{s,T}\left(X\right)+\int_{s}^{T}g\left(r,{\tilde{Y}}_{r}-Y_{s,T}\left(X\right),{\tilde{Z}}_{r},{\tilde{K}}_{r}\right)dr\\ & \;\;\;\;-\int_{s}^{T}{\tilde{Z}}_{r}dB_{r}-\sum_{i=1}^{\infty }\int_{s}^{T}{\tilde{K}}_{r}^{\left(i\right)}dH_{r}^{\left(i\right)}. \end{array}$$

Due to the uniqueness of the solution, we have

(30) $$\begin{array}{cc} Y_{t,T}\left(X\right) & =Y_{t,s}\left(X1_{\left[t,s\right)}-Y_{s,T}\left(X\right)1_{\left[s,s\right]}\right)\\ & =Y_{s,T}\left(X\right)+\int_{t}^{s}g\left(r,{\tilde{Y}}_{r}+X_{r},{\tilde{Z}}_{r},{\tilde{K}}_{r}\right)dr\\ & \;\;\;\;-\int_{t}^{s}{\tilde{Z}}_{r}dB_{r}-\sum_{i=1}^{\infty }\int_{t}^{s}{\tilde{K}}_{r}^{\left(i\right)}dH_{r}^{\left(i\right)}, \end{array}$$

(31) $$\begin{array}{cc} Y_{s,T}\left(-Y_{s,T}\left(X\right)1_{\left[s,T\right]}\right) & =Y_{s,T}\left(X\right)+\int_{s}^{T}g\left(r,{\tilde{Y}}_{r}-Y_{s,T}\left(X\right),{\tilde{Z}}_{r},{\tilde{K}}_{r}\right)dr\\ & \;\;\;\;-\int_{s}^{T}{\tilde{Z}}_{r}dB_{r}-\sum_{i=1}^{\infty }\int_{s}^{T}{\tilde{K}}_{r}^{\left(i\right)}dH_{r}^{\left(i\right)}. \end{array}$$

By Propositions 5 and 6(i), we get

(32) $$\begin{array}{cc} Y_{s,T}\left(-Y_{s,T}\left(X\right)1_{\left[s,T\right]}\right) & =Y_{s,T}\left(0-Y_{s,T}\left(X\right)1_{\left[s,T\right]}\right)\\ \; & =Y_{s,T}\left(0\right)+Y_{s,T}\left(X\right)\\ \; & =Y_{s,T}\left(X\right). \end{array}$$

Finally, we have

$$\begin{array}{cc} Y_{t}\left(X1_{\left[t,s\right)}-Y_{s}\left(X\right)1_{\left[s,T\right]}\right) & =Y_{t}\left(\tilde{X}\right)={\tilde{Y}}_{t}\\ & =Y_{t}\left(X\right)-Y_{s,T}\left(X\right)+Y_{s,T}\left(X\right)\\ & =Y_{t}\left(X\right), \end{array}$$

for all $X\in S_{T}^{2},t\in \left[0,T\right],s\in \left[t,T\right]$.  □

For conditional convexity of $Y_{t}\left(\cdot \right)$, we have the following result.

**Proposition** **7.** *Assume that g satisfies (H1), (H2), and (H3). Denote by $(Y_{t}\left(X\right),Z_{t}\left(X\right),$$K_{t}\left(X\right))$ the solution of BSDEL (6) corresponding to $X\in S_{T}^{2}$. Then, the following statements are equivalent:*

*(i)*  *For any t∈[0,T], $Y_{t}\left(\cdot \right)$ is conditional convex in $S_{T}^{2}$, i.e., for each $X^{1},X^{2}\in S_{T}^{2},\lambda \in L^{2}\left(\Omega ,F_{t},P\right),\lambda \in \left[0,1\right]$,*
  $$Y_{t}\left(\lambda X^{1}+\left(1-\lambda \right)X^{2}\right)\leq \lambda Y_{t}\left(X^{1}\right)+\left(1-\lambda \right)Y_{t}\left(X^{2}\right),\;\;\;\;\;\;a.s.$$
*(ii)*  *For any t∈[0,T], $Y_{t}\left(\cdot \right)$ is conditional convex in $R^{\infty }$, i.e., for each $X^{1},X^{2}\in R^{\infty },\lambda \in L^{\infty }\left(\Omega ,F_{t},P\right),\lambda \in \left[0,1\right]$,*
  $$Y_{t}\left(\lambda X^{1}+\left(1-\lambda \right)X^{2}\right)\leq \lambda Y_{t}\left(X^{1}\right)+\left(1-\lambda \right)Y_{t}\left(X^{2}\right),\;\;\;\;\;\;a.s.$$
*(iii)*  *g satisfies (H4), i.e., g is convex in (y,z,k).*

**Proof.**  First, we prove that (iii) ⇒ (i) holds. Suppose that *g* satisfies (H4). Let $X^{1},X^{2}\in S_{T}^{2}$. Denote by $(Y_{t}^{1}\left(X\right),Z_{t}^{1}\left(X\right),$ $K_{t}^{1}\left(X\right))$ and $(Y_{t}^{2}\left(X\right),Z_{t}^{2}\left(X\right),$ $K_{t}^{2}\left(X\right))$ the solutions of BSDEL (6) corresponding to $X=X^{1}$ and $X=X^{2}$, respectively. Then, we have

$$\begin{array}{c} Y_{t}^{1}=-X_{T}^{1}+\int_{t}^{T}g\left(s,Y_{s}^{1}+X_{s}^{1},Z_{s}^{1},K_{s}^{1}\right)ds-\int_{t}^{T}Z_{s}^{1}dB_{s}-\sum_{i=1}^{\infty }\int_{t}^{T}K_{s}^{1(i)}dH_{s}^{\left(i\right)},\;\;\;t\in \left[0,T\right], \end{array}$$

$$\begin{array}{c} Y_{t}^{2}=-X_{T}^{2}+\int_{t}^{T}g\left(s,Y_{s}^{2}+X_{s}^{2},Z_{s}^{2},K_{s}^{2}\right)ds-\int_{t}^{T}Z_{s}^{2}dB_{s}-\sum_{i=1}^{\infty }\int_{t}^{T}K_{s}^{2(i)}dH_{s}^{\left(i\right)},\;\;\;t\in \left[0,T\right]. \end{array}$$

For all $\lambda \in L^{2}\left(\Omega ,F_{t},P\right)$ and λ∈[0,1], we set

$$\tilde{X}=\lambda X^{1}+\left(1-\lambda \right)X^{2},\;\tilde{Y}=\lambda Y^{1}+\left(1-\lambda \right)Y^{2},\;\tilde{Z}=\lambda Z^{1}+\left(1-\lambda \right)Z^{2},\;\tilde{K}=\lambda K^{1}+\left(1-\lambda \right)K^{2}.$$

By assumption (H4), we get

$$\begin{array}{c} g\left(t,{\tilde{Y}}_{t}+{\tilde{X}}_{t},{\tilde{Z}}_{t},{\tilde{K}}_{t}\right)\leq \lambda g\left(t,Y_{t}^{1}+X_{t}^{1},Z_{t}^{1},K_{t}^{1}\right)+\left(1-\lambda \right)g\left(t,Y_{t}^{2}+X_{t}^{2},Z_{t}^{2},K_{t}^{2}\right),\;\;\;a.s. \end{array}$$

Thus, we have

$$\begin{array}{cc} & \lambda Y_{t}\left(X^{1}\right)+\left(1-\lambda \right)Y_{t}\left(X^{2}\right)\\ & =-\left(\lambda X_{T}^{1}+\left(1-\lambda \right)X_{T}^{2}\right)+\int_{t}^{T}\lambda g\left(s,Y_{s}^{1}+X_{s}^{1},Z_{s}^{1},K_{s}^{1}\right)ds+\int_{t}^{T}\left(1-\lambda \right)g\left(s,Y_{s}^{2}+X_{s}^{2},Z_{s}^{2},K_{s}^{2}\right)ds\\ & \;\;\;\;-\int_{t}^{T}\left(\lambda Z_{s}^{1}+\left(1-\lambda \right)Z_{s}^{2}\right)dB_{s}-\sum_{i=1}^{\infty }\int_{t}^{T}\left(\lambda K_{s}^{1(i)}+\left(1-\lambda \right)K_{s}^{2(i)}\right)dH_{s}^{\left(i\right)} \end{array}$$

(33) $$\begin{array}{cc} \; & =-{\tilde{X}}_{T}\;+\;\int_{t}^{T}\left(\lambda g\left(s,Y_{s}^{1}+X_{s}^{1},Z_{s}^{1},K_{s}^{1}\right)\;+\;\left(1-\lambda \right)g\left(s,Y_{s}^{2}+X_{s}^{2},Z_{s}^{2},K_{s}^{2}\right)\right)ds\\ \; & \;\;\;\;-\int_{t}^{T}{\tilde{Z}}_{s}dB_{s}-\sum_{i=1}^{\infty }\int_{t}^{T}{\tilde{K}}_{s}dH_{s}^{\left(i\right)}\\ \; & \geq -{\tilde{X}}_{T}+\int_{t}^{T}g\left(s,{\tilde{Y}}_{s}+{\tilde{X}}_{s},{\tilde{Z}}_{s},{\tilde{K}}_{s}\right)ds-\int_{t}^{T}{\tilde{Z}}_{s}dB_{s}-\sum_{i=1}^{\infty }\int_{t}^{T}{\tilde{K}}_{s}^{\left(i\right)}dH_{s}^{\left(i\right)}. \end{array}$$

Note that ${\tilde{Y}}_{t}=\lambda Y_{t}\left(X^{1}\right)+\left(1-\lambda \right)Y_{t}\left(X^{2}\right)$. By Proposition 2, we get for all $X^{1},X^{2}\in S_{T}^{2}$, $\lambda \in L^{2}\left(\Omega ,F_{t},P\right),\lambda \in \left[0,1\right],t\in \left[0,T\right],$

$$Y_{t}\left(\lambda X^{1}+\left(1-\lambda \right)X^{2}\right)\leq \lambda Y_{t}\left(X^{1}\right)+\left(1-\lambda \right)Y_{t}\left(X^{2}\right),\;\;\;\;\;\;a.s.$$

Second, let us prove that (i) ⇒ (iii) holds. For each $\xi ,\eta \in L^{2}\left(\Omega ,F_{T},P\right)$, we set

$$X^{1}=\xi 1_{\left[T,T\right]},\;X^{2}=\eta 1_{\left[T,T\right]}.$$

Then, $X^{1},X^{2}\in S_{T}^{2}$. Consider the following BSDELs:

$$\begin{array}{c} Y_{t}^{1}=-\xi +\int_{t}^{T}g\left(r,Y_{r}^{1},Z_{r}^{1},K_{r}^{1}\right)dr-\int_{t}^{T}Z_{r}^{1}dB_{r}-\sum_{i=1}^{\infty }\int_{t}^{T}K_{r}^{1(i)}dH_{r}^{\left(i\right)},\;\;\;t\in \left[0,T\right], \end{array}$$

$$\begin{array}{c} Y_{t}^{2}=-\eta +\int_{t}^{T}g\left(r,Y_{r}^{2},Z_{r}^{2},K_{r}^{2}\right)dr-\int_{t}^{T}Z_{r}^{2}dB_{r}-\sum_{i=1}^{\infty }\int_{t}^{T}K_{r}^{2(i)}dH_{r}^{\left(i\right)},\;\;\;t\in \left[0,T\right]. \end{array}$$

By the uniqueness of solutions of BSDELs, we get

$$Y_{t}\left(X^{1}\right)=Y_{t}^{1}=Y_{t}\left(g,T,-\xi \right),\;\;\;\;Y_{t}\left(X^{2}\right)=Y_{t}^{2}=Y_{t}\left(g,T,-\eta \right).$$

Then, by using the conditional convexity of $Y_{t}\left(\cdot \right)$, we have for each $\xi ,\eta \in L^{2}\left(\Omega ,F_{T},P\right),\lambda \in \left[0,1\right],t\in \left[0,T\right]$,

(34) $$\begin{array}{c} Y_{t}\left(g,T,\lambda \xi +(1-\lambda )\eta \right)\leq \lambda Y_{t}\left(g,T,\xi \right)+\left(1-\lambda \right)Y_{t}\left(g,T,\eta \right),\;\;\;a.s. \end{array}$$

For all $\left(y_{1},z_{1},k_{1}\right),\left(y_{2},z_{2},k_{2}\right)\in R\times R\times \ell^{2},\lambda \in \left[0,1\right]$, let

$$\tilde{y}=\lambda y_{1}+\left(1-\lambda \right)y_{2},\;\;\tilde{z}=\lambda z_{1}+\left(1-\lambda \right)z_{2},\;\;\tilde{k}=\lambda k_{1}+\left(1-\lambda \right)k_{2}.$$

Using Theorem 1, we get that there exists a subsequence ${\left\{n_{m}\right\}}_{m=1}^{\infty }\subset {\left\{n\right\}}_{n=1}^{\infty }$ such that dP×dt-a.s.,

(35) $$\begin{array}{cc} g(t,\tilde{y},\tilde{z},\tilde{k}) & =\lim_{m\to \infty }n_{m}[Y_{t}(g,t+\frac{1}{n_{m}},\tilde{y}+\tilde{z}\left(B_{t+\frac{1}{n_{m}}}-B_{t}\right)\\ \; & \;\;\;\;\left.\left.+\sum_{i=1}^{\infty }{\tilde{k}}_{i}\left(H_{t+\frac{1}{n_{m}}}^{\left(i\right)}-H_{t}^{\left(i\right)}\right)\right)-\tilde{y}\right]. \end{array}$$

Using Theorem 1 again, we get

(36) $$\begin{array}{cc} g(t,y_{1},z_{1},k_{1}) & =L^{p}-\lim_{m\to \infty }n_{m}[Y_{t}(g,t+\frac{1}{n_{m}},y_{1}+z_{1}\left(B_{t+\frac{1}{n_{m}}}-B_{t}\right)\\ \; & \;\;\;\;\left.\left.+\sum_{i=1}^{\infty }k_{1,i}\left(H_{t+\frac{1}{n_{m}}}^{\left(i\right)}-H_{t}^{\left(i\right)}\right)\right)-y_{1}\right]. \end{array}$$

Furthermore, there exists a subsequence ${\left\{n_{mj}\right\}}_{j=1}^{\infty }\subset {\left\{n_{m}\right\}}_{m=1}^{\infty }$ such that dP×dt-a.s.,

(37) $$\begin{array}{cc} g\left(t,y_{1},z_{1},k_{1}\right) & =\lim_{j\to \infty }n_{mj}[Y_{t}(g,t+\frac{1}{n_{mj}},y_{1}+z_{1}\left(B_{t+\frac{1}{n_{mj}}}-B_{t}\right)\\ \; & \;\;\;\;\left.\left.+\sum_{i=1}^{\infty }k_{1,i}\left(H_{t+\frac{1}{n_{mj}}}^{\left(i\right)}-H_{t}^{\left(i\right)}\right)\right)-y_{1}\right]. \end{array}$$

Similarly, there exists a subsequence ${\left\{n_{mjl}\right\}}_{l=1}^{\infty }\subset {\left\{n_{mj}\right\}}_{j=1}^{\infty }$ such that dP×dt-a.s.,

(38) $$\begin{array}{cc} g\left(t,y_{2},z_{2},k_{2}\right) & =\lim_{l\to \infty }n_{mjl}[Y_{t}(g,t+\frac{1}{n_{mjl}},y_{2}+z_{2}\left(B_{t+\frac{1}{n_{mjl}}}-B_{t}\right)\\ \; & \;\;\;\;\left.\left.+\sum_{i=1}^{\infty }k_{2,i}\left(H_{t+\frac{1}{n_{mjl}}}^{\left(i\right)}-H_{t}^{\left(i\right)}\right)\right)-y_{2}\right]. \end{array}$$

Due to the uniqueness of the solution and assumption (H3), for all s∈[0,T], $\xi \in L^{2}\left(\Omega ,F_{s},P\right)$, we get

(39) $$\begin{array}{c} Y_{t}\left(g,T,\xi \right)=Y_{t}\left(g,s,\xi \right),\;\;\;\forall t\in \left[0,s\right] \end{array}$$

Thus, combining (34) and (39), for all $\left(y_{1},z_{1},k_{1}\right),\left(y_{2},z_{2},k_{2}\right)\in R\times R\times \ell^{2},\lambda \in \left[0,1\right]$, we have

(40) $$\begin{array}{cc} \; & Y_{t}\left(g,t\;+\;\frac{1}{n_{mjl}},\lambda y_{1}\;+\;\left(1\;-\;\lambda \right)y_{2}+\left(\lambda z_{1}\;+\;\left(1-\lambda \right)z_{2}\right)\left(B_{t+\frac{1}{n_{mjl}}}\;-\;B_{t}\right)\right.\\ \; & \;\;\;\;\left.+\sum_{i=1}^{\infty }\left(\lambda k_{1,i}\;+\;\left(1\;-\;\lambda \right)k_{2,i}\right)\left(H_{t+\frac{1}{n_{mjl}}}^{\left(i\right)}-H_{t}^{\left(i\right)}\right)\right)\;-\left(\;\lambda y_{1}+\left(1\;-\;\lambda \right)y_{2}\right)\\ \; & \leq \lambda \left(Y_{t}\left(g,t+\frac{1}{n_{mjl}},y_{1}+z_{1}\left(B_{t+\frac{1}{n_{mjl}}}-B_{t}\right)+\sum_{i=1}^{\infty }k_{1,i}\left(H_{t+\frac{1}{n_{mjl}}}^{\left(i\right)}-H_{t}^{\left(i\right)}\right)\right)-y_{1}\right)\\ \; & \;\;\;\;+\left(1-\lambda \right)\left(Y_{t}\left(g,t+\frac{1}{n_{mjl}},y_{2}+z_{2}\left(B_{t+\frac{1}{n_{mjl}}}-B_{t}\right)+\sum_{i=1}^{\infty }k_{2,i}\left(H_{t+\frac{1}{n_{mjl}}}^{\left(i\right)}-H_{t}^{\left(i\right)}\right)\right)-y_{2}\right). \end{array}$$

Thus, using Theorem 1, we deduce that for all $\left(y_{1},z_{1},k_{1}\right),\left(y_{2},z_{2},k_{2}\right)\in R\times R\times \ell^{2}$, λ∈[0,1],

$g\left(t,\lambda y_{1}+\left(1-\lambda \right)y_{2},\lambda z_{1}+\left(1-\lambda \right)z_{2},\lambda k_{1}+\left(1-\lambda \right)k_{2}\right)$

$$\;\;\;\;\;\;\;\;\;\;\;\;\;\;\;\;\;\;\;\;\leq \lambda g\left(t,y_{1},z_{1},k_{1}\right)+\left(1-\lambda \right)g\left(t,y_{2},z_{2},k_{2}\right),\;\;\;\;\;dP\times dt-a.s.$$

That is, *g* satisfies assumption (H4).

Obviously, (iii) ⇒(i) implies (iii) ⇒ (ii). Finally, we prove that (ii) ⇒ (iii) holds. Suppose that $Y_{t}\left(\cdot \right)$ is conditional convex in $R^{\infty }$. Then, we get

(41) $$\begin{array}{c} Y_{t}\left(g,T,\lambda \xi_{n}+\left(1-\lambda \right)\eta_{n}\right)\leq \lambda Y_{t}\left(g,T,\xi_{n}\right)+\left(1-\lambda \right)Y_{t}\left(g,T,\eta_{n}\right),\;\;\;a.s., \end{array}$$

for all $\xi_{n},\eta_{n}\in L^{\infty }\left(\Omega ,F_{T},P\right),\lambda \in \left[0,1\right],t\in \left[0,T\right].$

Let

$$\xi_{n}=\xi 1_{|\xi |\leq n},\;\eta_{n}=\xi 1_{|\eta |\leq n},\;\;\;\;\;\;\;\xi ,\eta \in L^{2}\left(\Omega ,F_{T},P\right).$$

Then, we have $\xi_{n},\eta_{n}\in L^{\infty }\left(\Omega ,F_{T},P\right)$ and $\xi_{n}\to \xi ,\eta_{n}\to \eta$ in $L^{2}$ sense. With the help of Proposition 1 and the similar argument as in (35)–(38), we deduce that (34) holds. By using assumption (H3) and Proposition 1, we also obtain that (39) holds. Thus, with the same method as in the proof of (i) ⇒ (iii), we see that *g* satisfies assumption (H4).  □

Following the similar argument of conditional convexity of $Y_{t}\left(\cdot \right)$ in Proposition 7, we get the following proposition.

**Proposition** **8.** *Assume that g satisfies (H1), (H2), and (H3). Denote by $(Y_{t}\left(X\right),Z_{t}\left(X\right),$$K_{t}\left(X\right))$ the solution of BSDEL (6) corresponding to $X\in S_{T}^{2}$. Then, the following statements are equivalent:*

*(i)*  *For any t∈[0,T], $Y_{t}\left(\cdot \right)$ is subadditive in $S_{T}^{2}$, i.e., for each $X^{1},X^{2}\in S_{T}^{2}$,*
  $$Y_{t}\left(X^{1}+X^{2}\right)\leq Y_{t}\left(X^{1}\right)+Y_{t}\left(X^{2}\right),\;\;\;\;\;\;a.s.$$
*(ii)*  *For any t∈[0,T], $Y_{t}\left(\cdot \right)$ is subadditive in $R^{\infty }$, i.e., for each $X^{1},X^{2}\in R^{\infty }$,*
  $$Y_{t}\left(X^{1}+X^{2}\right)\leq Y_{t}\left(X^{1}\right)+Y_{t}\left(X^{2}\right),\;\;\;\;\;\;a.s.$$
*(iii)*  *g satisfies (H5), i.e., g is subadditive in (y,z,k).*

For monotonicity of $Y_{t}\left(\cdot \right)$, we have the following result.

**Proposition** **9.** *Assume that g satisfies (H1), (H2), and (H3). Denote by $(Y_{t}\left(X\right),Z_{t}\left(X\right),$$K_{t}\left(X\right))$ the solution of BSDEL (6) corresponding to $X\in S_{T}^{2}$. Then, the following statements are equivalent:*

*(i)*  *For each $X^{1},X^{2}\in S_{T}^{2},t\in \left[0,T\right]$, $Y_{t}\left(X^{1}\right)\leq Y_{t}\left(X^{2}\right)$, if $X^{1}\geq X^{2}$.*
*(ii)*  *For each $X^{1},X^{2}\in R^{\infty },t\in \left[0,T\right]$, $Y_{t}\left(X^{1}\right)\leq Y_{t}\left(X^{2}\right)$, if $X^{1}\geq X^{2}$.*
*(iii)*  *g satisfies (H7), i.e., g is nonincreasing in y.*

**Proof.**  First, we prove that (iii) ⇒ (i) holds. Let $X^{1},X^{2}\in S_{T}^{2}$, and $X^{1}\geq X^{2}$. Then, we have $-X^{1}\left(T\right)\leq -X^{2}\left(T\right)$ and $y+X^{1}\geq y+X^{2},\forall y\in R,t\in \left[0,T\right]$. Notice that g is nonincreasing in *y*. We have

$$\begin{array}{c} f^{X^{1}}\left(t,y,z,k\right):=g\left(t,y+X^{1}\left(t\right),z,k\right)\leq g\left(t,y+X^{2}\left(t\right),z,k\right):=f^{X^{2}}\left(t,y,z,k\right), \end{array}$$

for all $\left(y,z,k\right)\in R\times R\times \ell^{2}$. By Proposition 2, we get $Y_{t}\left(X^{1}\right)\leq Y_{t}\left(X^{2}\right).$

Second, we prove that (i) ⇒ (iii) holds. Let

$$X^{1}=a1_{\left[0,T\right)}-\xi 1_{\left[T,T\right]},\;\;\;\;\;X^{2}=b1_{\left[0,T\right)}-\xi 1_{\left[T,T\right]},$$

where a,b∈R,a≥b and $\xi \in L^{2}\left(\Omega ,F_{T},P\right)$. Then, we have $X^{1},X^{2}\in S_{T}^{2}$ and $X^{1}\geq X^{2}$.

Suppose that (i) holds, For any $\xi \in L^{2}\left(\Omega ,F_{T},P\right)$, we have

(42) $$\begin{array}{c} Y_{t}\left(f^{X^{1}},T,\xi \right)\leq Y_{t}\left(f^{X^{2}},T,\xi \right),\;\;\;\;\;\;\;t\in \left[0,T\right]. \end{array}$$

Similar to obtaining (39), due to the uniqueness of the solution of BSDEL and assumption (H3), we get

(43) $$\begin{array}{c} Y_{t}\left(f^{X^{i}},T,\xi \right)=Y_{t}\left(f^{X^{i}},s,\xi \right),\;\;\;\;\;\;i=1,2, \end{array}$$

for all $\xi \in L^{2}\left(\Omega ,F_{s},P\right),s\in \left[0,T\right],t\in \left[0,s\right]$. With the help of Theorem 1 and the similar argument as in (35)–(38), we have for all $\left(y,z,k\right)\in R\times R\times \ell^{2},t\in \left[0,T\right]$,

g(t,y+a,z,k)≤g(t,y+b,z,k),a.s.

Notice that the choice of *a* and *b* is arbitrary and a≥b, we have that g is nonincreasing in *y*.

Obviously, (iii) ⇒ (i) implies (iii) ⇒ (ii). Finally, we prove that (ii) ⇒ (iii) holds. Suppose that (ii) holds. Let

$$X_{n}^{1}=a1_{\left[0,T\right)}-\xi_{n}1_{\left[T,T\right]},\;\;\;\;\;X_{n}^{2}=b1_{\left[0,T\right)}-\xi_{n}1_{\left[T,T\right]},$$

where a,b∈R,a≥b and $\xi \in L^{\infty }\left(\Omega ,F_{T},P\right)$. Then we have $X_{n}^{1},X_{n}^{2}\in R^{\infty }$ and $Y_{t}\left(X_{n}^{1}\right)\leq Y_{t}\left(X_{n}^{2}\right),t\in \left[0,T\right]$. Using Proposition 1 and the similar argument as in (35)–(38), we have

$$Y_{t}\left(X^{1}\right)\leq Y_{t}\left(X^{2}\right),\;\;\;\;t\in \left[0,T\right],$$

where $X^{1}=a1_{\left[0,T\right)}-\xi 1_{\left[T,T\right]},X^{2}=b1_{\left[0,T\right)}-\xi 1_{\left[T,T\right]},\xi \in L^{2}\left(\Omega ,F_{T},P\right).$ Thus, by using Proposition 1, Theorem 1, and the same method as in the proof of (i) ⇒ (iii), we can see that *g* satisfies assumption (H7).  □

For conditional positive homogeneity of $Y_{t}\left(\cdot \right)$, we have the following result.

**Proposition** **10.** *Assume that g satisfies (H1) and (H2). Denote by $(Y_{t}\left(X\right),Z_{t}\left(X\right),$$K_{t}\left(X\right))$ the solution of BSDEL (6) corresponding to $X\in S_{T}^{2}$. Then, the following statements are equivalent:*

*(i)*  *For any t∈[0,T], $Y_{t}\left(\cdot \right)$ is positively homogeneous in $S_{T}^{2}$, i.e., for each $X\in S_{T}^{2},\lambda \in L^{\infty }\left(\Omega ,F_{t},P\right),\lambda \geq 0$,*
  $$Y_{t}\left(\lambda X\right)=\lambda Y_{t}\left(X\right),\;\;\;\;\;\;a.s.$$
*(ii)*  *For any t∈[0,T], $Y_{t}\left(\cdot \right)$ is positively homogeneous in $R^{\infty }$, i.e., for each $X\in R^{\infty },\lambda \in L^{\infty }\left(\Omega ,F_{t},P\right),\lambda \geq 0$,*
  $$Y_{t}\left(\lambda X\right)=\lambda Y_{t}\left(X\right),\;\;\;\;\;\;a.s.$$
*(iii)*  *g satisfies (H6), i.e., g is positively homogeneous in (y,z,k).*

**Proof.**  First, we prove that (iii) ⇒ (i) holds. Let $X^{1}\in S_{T}^{2},t\in \left[0,T\right],\lambda \in L^{\infty }\left(\Omega ,F_{t},P\right)$, λ≥0 and $X^{2}=\lambda X^{1}$. Then, we have $X^{2}\in S_{T}^{2}$. Let us denote by $\left(Y^{1},Z^{1},K^{1}\right)$ and $\left(Y^{2},Z^{2},K^{2}\right)$ the adapted solution of BSDEL (6) corresponding to $X=X^{1}$ and $X=X^{2}$, respectively. Notice that *g* satisfies the assumption (H6), that is, *g* is positively homogeneous in (y,z,k). Then, we have

$$\begin{array}{cc} \lambda Y_{t}^{1} & =-\lambda X_{T}^{1}+\int_{t}^{T}g\left(s,\lambda Y_{s}^{1}+\lambda X_{s}^{1},\lambda Z_{s}^{1},\lambda K_{s}^{1}\right)ds-\int_{t}^{T}Z_{s}^{1}dB_{s}\\ & \;\;\;\;-\sum_{i=1}^{\infty }\int_{t}^{T}K_{s}^{1(i)}dH_{s}^{\left(i\right)},\;\;\;t\in \left[0,T\right], \end{array}$$

$$\begin{array}{cc} Y_{t}^{2} & =-\lambda X_{T}^{1}+\int_{t}^{T}g\left(s,Y_{s}^{2}+\lambda X_{s}^{1},Z_{s}^{2},K_{s}^{2}\right)ds-\int_{t}^{T}Z_{s}^{2}dB_{s}\\ & \;\;\;\;-\sum_{i=1}^{\infty }\int_{t}^{T}K_{s}^{2(i)}dH_{s}^{\left(i\right)},\;\;\;t\in \left[0,T\right]. \end{array}$$

Due to uniqueness of the solution, we get for any $X\in S_{T}^{2},\lambda \in L^{\infty }\left(\Omega ,F_{t},P\right)$, λ≥0,t∈[0,T],

$$Y_{t}\left(\lambda X\right)=Y_{t}^{2}=\lambda Y_{t}^{1}=\lambda Y_{t}\left(X\right).\;$$

Second, we prove that (i) ⇒ (iii) holds. Suppose that $Y_{t}\left(\cdot \right)$ is positively homogeneous in $S_{T}^{2}$. We obtain $Y_{t}\left(0\right)=0$ for all t∈[0,T]. By proposition 5, we know that *g* satisfies (H3). Due to uniqueness of the solution and assumption (H3), we also get

$$Y_{t}\left(g,T,\xi \right)=Y_{t}\left(g,s,\xi \right),$$

for all s∈[0,T],t∈[0,s], $\xi \in L^{2}\left(\Omega ,F_{s},P\right)$. Applying the positive homogeneity of $Y_{t}\left(\cdot \right)$ in $S_{T}^{2}$, for all $\xi \in L^{2}\left(\Omega ,F_{T},P\right),\alpha \geq 0,$ we have

$$Y_{t}\left(g,T,\alpha \xi \right)=\alpha Y_{t}\left(g,T,\xi \right).\;\;\;$$

With the help of Lemma 1 and the similar argument as in (35)–(38), we have for any $\left(y,z,k\right)\in R^{2}\times \ell^{2}$, α≥0,

$$g\left(t,\alpha y,\alpha z,\alpha k\right)=\alpha g\left(t,y,z,k\right),\;\;\;\;dP\times dt-a.s.$$

That is, *g* satisfies assumption (H6).

Obviously, (iii) ⇒ (i) implies (iii) ⇒ (ii). Finally, we prove that (ii) ⇒ (iii) holds. Suppose that (ii) holds. Then, we have

$$Y_{t}\left(g,T,\alpha \xi_{n}\right)=\alpha Y_{t}\left(g,T,\xi_{n}\right),$$

for any $\xi_{n}\in L^{2}\left(\Omega ,F_{T},P\right),t\in \left[0,T\right],\alpha \geq 0.$

Applying the positive homogeneity of $Y_{t}\left(\cdot \right)$ in $R^{\infty }$, we obtain $Y_{t}\left(0\right)=0$ for all t∈[0,T]. Let $\xi_{n}=\xi 1_{|\xi |\leq n}$, where $\xi \in L^{2}\left(\Omega ,F_{T},P\right)$. Then, we have $\xi_{n}\in L^{\infty }\left(\Omega ,F_{T},P\right)$ and $\xi_{n}\to \xi$ in $L^{2}$ sense. Using Theorem 1, we get

$$Y_{t}\left(g,T,\alpha \xi \right)=\alpha Y_{t}\left(g,T,\xi \right),\;\;\;$$

for any $\xi \in L^{2}\left(\Omega ,F_{T},P\right),t\in \left[0,T\right],\alpha \geq 0.$ With the help of the same method as in the proof of (i) ⇒ (iii), we can see that *g* satisfies assumption (H6).  □

**Proof** **of** **Theorem** **2.** Using Propositions 5, 6(i), 7, and 9, we can directly see that (i) is right. Furthermore, (ii) is implied by Theorem 2(i) and Proposition 6(ii).  □

**Proof** **of** **Theorem** **3.** Using Propositions 5, 6(i), and 8–10, we can directly see that (i) is right. Furthermore, (ii) is implied by Theorem 3(i) and Proposition 6(ii).  □

## 5. Numerical Illustrations

In this section, we will provide two numerical examples to illustrate the proposed dynamic risk measures.

**Example** **1.** *We suppose that the generator of the BSDEL (6) is independent of (y,k) and is given by*

$$g\left(z\right)=\left\{\begin{array}{cc} z^{2},\;\; & |z|\leq 1,\\ 2|z|-1,\;\; & |z|>1. \end{array}\right.$$

*Let $X_{t}=\sigma^{2}t-\sigma B_{t},t\in \left[0,T\right]$, σ∈(0,1]. For any $X\in S_{T}^{2}$ and σ∈(0,1], we consider the following equation:*

(44) $$\begin{array}{c} Y_{t}=-X_{T}+\int_{t}^{T}g\left(Z_{s}\right)ds-\int_{t}^{T}Z_{s}dB_{s},\;\;\;t\in \left[0,T\right]. \end{array}$$

The solution of (44) is

$$\left(Y_{t},Z_{t}\right)=\left(-\sigma^{2}t+\sigma B_{t},\sigma \right),\;\;\;\;\;t\in \left[0,T\right].$$

Let

$$\rho_{t}\left(X\right)=-\sigma^{2}t+\sigma B_{t},\;\;\;\;\forall t\in \left[0,T\right],X\in R^{\infty }.$$

By Theorem 2, we obtain that ρ is a dynamic convex risk measure.

**Figure 1:**
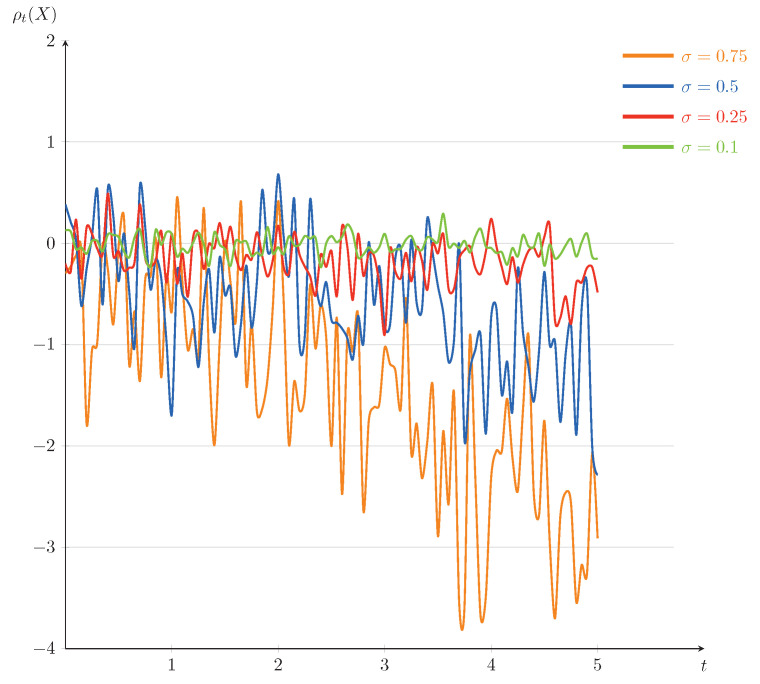
The trends of $\rho_{t}\left(X\right)$ as a funtion of *t* for Example 1 (fixed σ=0.1,0.25,0.5,0.75).

**Figure 2:**
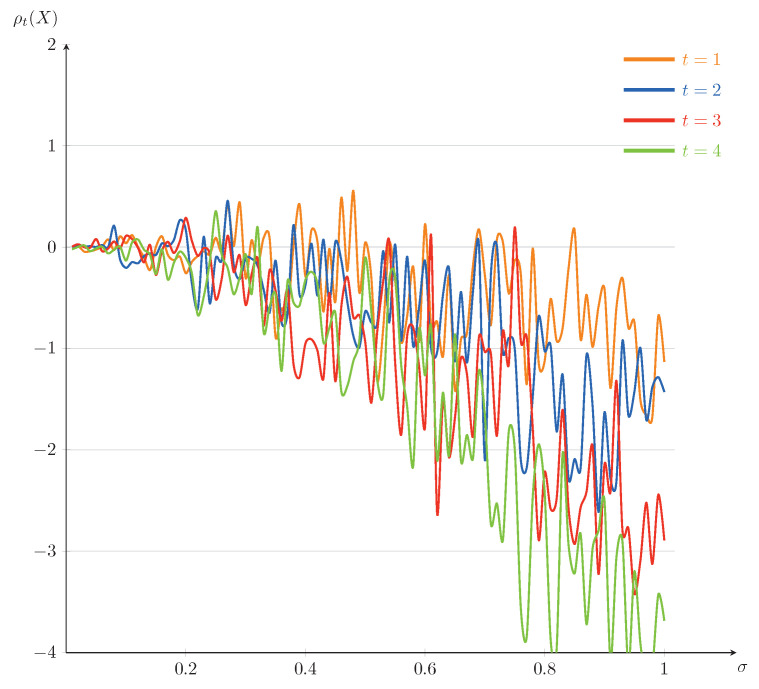
The trends of $\rho_{t}\left(X\right)$ as a funtion of σ for Example 1 (fixed t=1,2,3,4).

In the following, we will present some numerical illustrations for this example. Set T=5. The curves of $\rho_{t}\left(X\right)$ as a function of *t* (for σ=0.1,0.25,0.5,0.75) and as a function of σ (for t=1,2,3,4) are plotted in Figure 1 and Figure 2, respectively. From Figure 1 and Figure 2, it is interesting to note that the dynamic risk measures ρ tend to decline on the whole, which is consistent with our intuitive understanding: in securities trading, when the stock price drops, the loss of investors increases and the corresponding cost risk also increases, as a result, the absolute value of the dynamic risk measure becomes larger. Furthermore, we find that the values of the dynamic risk measures ρ appear positive on some time interval, which can be interpreted by the effect of the large disturbance of Brownian motion at some point. We mention that the fluctuations of the dynamic risk measures ρ become more stable in Figure 1 when σ becomes smaller, and the downward trends of the dynamic risk measures ρ become more obvious in Figure 2 when *t* becomes bigger. That is because of choosing to invest in low-risk assets and increasing of investment risk, respectively.

In a financial market, some investors may venture among certain European-type contingent claims, some bonds, some stocks, and so on. Depending on an investor’s appetite for risk, he/she may choose a curve in Figure 1 as their investment target, or choose a reasonable time of trading based on the impact of level of risk appetite in Figure 2. For example, in order to get more returns, a risk-lover may choose a curve of σ=0.75 in Figure 1 as his/her investment target in high-risk assets such as certain European-type contingent claims and some stocks. On the contrary, a risk-averse investor may choose a curve of σ=0.1 in Figure 1 as his/her investment target.

**Example** **2.** *We suppose that the generator of the BSDEL (6) is independent of (y,k) and is given by $g\left(z\right)=z^{2}.$ Let $X_{t}=\sigma^{2}t-\sigma B_{t},t\in \left[0,T\right]$, σ∈(0,1]. For any $X\in S_{T}^{2}$ and σ∈(0,1], we consider the following equation:*

(45) $$\begin{array}{c} Y_{t}=-\frac{1}{2}X_{T}+\int_{t}^{T}g\left(Z_{s}\right)ds-\int_{t}^{T}Z_{s}dB_{s},\;\;\;t\in \left[0,T\right]. \end{array}$$

The solution of (45) is

$$\left(Y_{t},Z_{t}\right)=\left(-\frac{\sigma^{2}}{4}\left(T+t\right)+\frac{\sigma }{2}B_{t},\frac{\sigma }{2}\right),\;\;\;\;\;t\in \left[0,T\right].$$

Let

$$\rho_{t}\left(X\right)=-\frac{\sigma^{2}}{4}\left(T+t\right)+\frac{\sigma }{2}B_{t},\;\;\;\;\forall t\in \left[0,T\right],X\in R^{\infty }.$$

From Theorem 2, we obtain that ρ is a dynamic convex risk measure.

**Figure 3:**
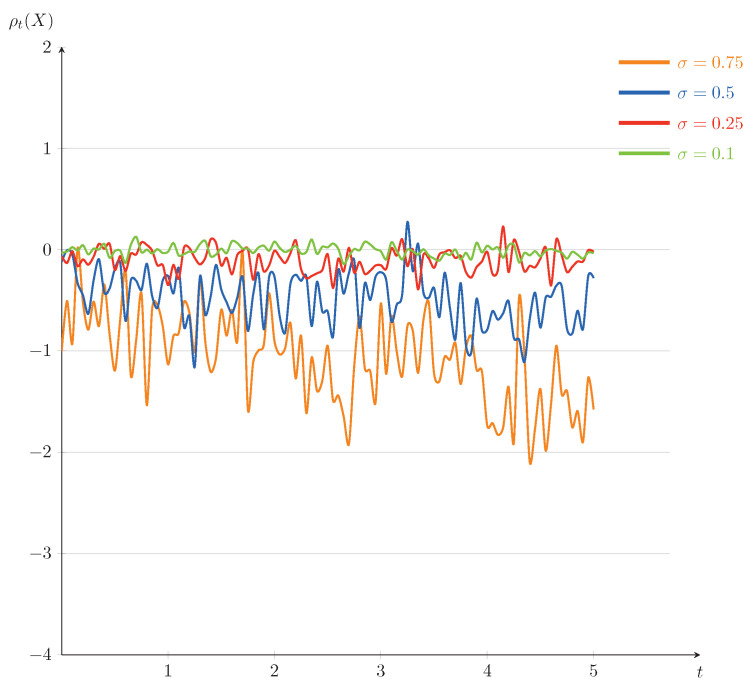
The trends of $\rho_{t}\left(X\right)$ as a function of *t* for Example 2 (fixed σ=0.1,0.25,0.5,0.75).

**Figure 4:**
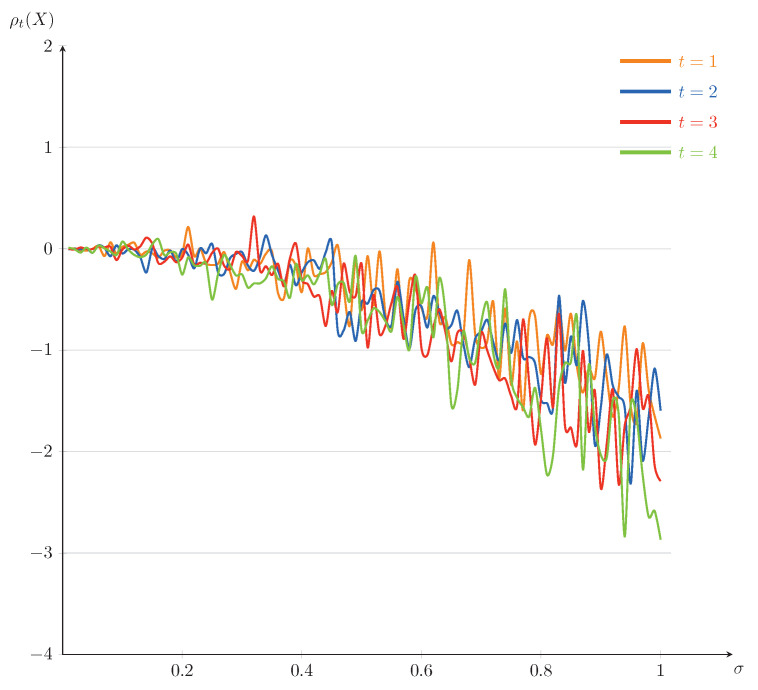
The trends of $\rho_{t}\left(X\right)$ as a function of σ for Example 2(fixed t=1,2,3,4).

In the following, we will also present some numerical illustrations for the example. Set T=5. The curves of $\rho_{t}\left(X\right)$ as a function of *t* (for σ=0.1,0.25,0.5,0.75) and as a function of σ (for t=1,2,3,4) are plotted in Figure 3 and Figure 4, respectively. It is interesting to note that, comparing with Figure 3, the downward trends of the dynamic risk measures ρ are clearly obvious in Figure 4. We mention that the fluctuations of the dynamic risk measures ρ become more stable in Figure 3 when σ becomes smaller, and there are no significant differences among the dynamic risk measures ρ in Figure 4 when *t* changes. Further, comparing Figure 3 and Figure 4 with Figure 1 and Figure 2, although the changing trends of the corresponding figures are similar, the fluctuation range of the former is smaller. This is because the solution of the current example is less affected by the diffusion term, which leads to a slower evolution speed than that of Example 1.

In a financial market, depending on investors’ appetite for risk, they may choose different investments in Figure 3. Figure 4 suggests that there may not be much difference for investors who choose a reasonable time of trading. Thus, the risk lovers, taking more risks, may choose the time t=4 of trading to get more returns.

## 6. Conclusions

In this paper, we study the dynamic risk measures for processes induced by backward stochastic differential equations driven by Teugel’s martingales associated with Lévy processes. The representation theorem for generators of BSDELs is provided. Furthermore, the time-consistency of the coherent and convex dynamic risk measures for processes is characterized by means of the generators of BSDELs. Moreover, the coherency and convexity of dynamic risk measures for processes are characterized by the generators of BSDELs. Finally, we provide two numerical examples to illustrate the proposed dynamic risk measures.
